# Neonatal vitamin A supplementation improves sheep fertility potential

**DOI:** 10.3389/fvets.2024.1370576

**Published:** 2024-05-02

**Authors:** Yating Li, Pengkang Song, Jiamin Zhao, Weipeng Zhang, Xiangdong Liu, Xiaoyang Lv, Junxing Zhao

**Affiliations:** ^1^College of Animal Science, Shanxi Agricultural University, Jinzhong, Shanxi, China; ^2^Department of Medical Oncology, Dana-Farber Cancer Institute, Boston, MA, United States; ^3^International Joint Research Laboratory in Universities of Jiangsu Province of China for Domestic Animal Germplasm Resources and Genetic Improvement, Yangzhou, Jiangsu, China

**Keywords:** sheep, vitamin A, testis, sperm, hormone, antioxidant

## Abstract

This study aimed to explore the effects of neonatal vitamin A (VA) supplementation on testis development and spermatogenesis. A total of 32 newborn lambs were intramuscularly injected with corn oil (control group) or corn oil + 2500 IU/kg BW VA (VA group). They were slaughtered and sampled at 3 weeks and 8 months of age to analyze spermatogenesis, cell proliferation, hormone secretion, antioxidant status of the testis, and adult sheep sperm parameters. Compared with the control group, the expression of spermatogonial differentiation-related genes in VA group was up-regulated (*P* < 0.05). Testis weight, seminiferous tubule diameter, number of spermatogonium and spermatocyte, and sperm density increased significantly in VA group at 8 months of age (*P* < 0.05). Neonatal VA injection upregulated the expression of the cell proliferation marker *PCNA* and cell cycle-related genes in the testis (*P* < 0.05). VA increased the concentrations of testosterone (T), luteinizing hormone (LH), and follicle-stimulating hormone (FSH) in the serum and upregulated steroidogenesis-related genes in the testis (*P* < 0.05). The antioxidant levels in the VA group were maintained at high levels. The total antioxidant capacity (T-AOC), antioxidant enzyme content and antioxidant-related genes were increased in the testis (*P* < 0.05). Furthermore, neonatal VA injection activated retinoic acid (RA) signaling to maintain the blood-testosterone barrier (BTB) in the testis of 3-week-old sheep. AMP-activated protein kinase (AMPK) and protein kinase B (AKT) signaling were also modulated in the sheep testis (*P* < 0.05). Taken together, VA supplementation in newborn rams promotes testis development and spermatogenesis to improve fertility.

## Introduction

Vitamin A (VA), also known as retinol, is a fat-soluble vitamin that is necessary in animals and is involved in various physiological activities such as vision maintenance, embryonic development, immune regulation, metabolism, organ development, and reproductive regulation (1). It can be considered a feed additive to improve the growth performance of livestock. VA deficiency leads to the inhibition of spermatogonial differentiation, spermatogonial apoptosis, vacuolation, calcification of spermatogenic epithelium, spermatogenic stasis, interruption of the blood-testis barrier (BTB), and degeneration and atrophy of the testis, resulting in sterility in male animals (2). VA supplementation restarts spermatogenesis and restores BTB levels in VA-deficient mice (3).

Retinoic acid (RA) is the active metabolite of VA. VA binds to retinol-binding protein to form a complex for transport in the blood circulation, then enters the cells via plasma membrane diffusion. VA undergoes a two-step enzymatic reaction in cells, in which it is first converted to retinal by alcohol dehydrogenase (ADH) and retinol dehydrogenase, the retinal is then converted to RA by retinal dehydrogenase (also known as ALDH). RA binds to cellular RA-binding protein in the nucleus and then binds with the retinoic acid receptor (RARs)/retinoic acid X receptor (RXRs) dimer to dissociate the RAR/RXR dimer from the retinoic acid reaction elements of the genome and co-suppressors. The inhibitory effect of RAR/RXR dimer on target genes is competitively blocked, and coactivators are recruited to induce the expression of RA-responsive genes (4–6). The RA receptors play a key role in spermatogenesis. The symptoms of targeted knockout of RARA are similar to those of VA-deficient mice, which also lead to pre-meiotic spermatocyte apoptosis and an abnormal spermatogenesis cycle (7).

Spermatogenesis involves the proliferation and differentiation of spermatogonial stem cells (SSCs), spermatocyte meiosis, and spermiation. First, A_single_ spermatogonia (A_s_) in the SSCs proliferates to form A_paired_ spermatogonia (A_pr_) and A_aligned_ spermatogonia (A_al_). A_al_ differentiates into A_1_-A_4_ spermatogonium, intermediate spermatogonium (In), type B spermatogonium and primary spermatocytes (pre-leptotene spermatocytes). Primary spermatocytes enter meiosis to form sperm cells that undergo morphological changes to form sperm. Immature sperm is released from the Sertoli cells into the lumen and through the testicular reticulum into the epididymis after spermatogenesis is complete. Germ cells at different stages in the testis are arranged successively from the basal to the apical compartment of seminiferous tubules and make up the seminiferous epithelium with Sertoli cells (8, 9). There is a BTB layer between the basal and apical compartments of the seminiferous epithelium. Spermatogonocytes develop into preleptotene spermatocytes and pass through the BTB for further meiosis. The BTB isolates germ cells of the basal compartment from the vasculature; therefore, meiosis is regulated periodically by the spermatogenic epithelium without interference from external factors (10, 11).

RA is a key factor in inducing SSC differentiation and initiating meiosis in males (12–14). However, synergistic regulation among multiple cell types in the testis and its regulatory mechanisms in downstream signaling pathways remain unclear. Moreover, the period after birth is the peak of various tissue and organ development in the body of animals, and the health status of a newborn continues to affect its entire life. Therefore, this study aimed to investigate the effects of neonatal VA supplementation on male animal fertility. The expression of cell proliferation-, steroidogenesis-, and antioxidation-related genes and the activity of key signaling pathways in the testes were determined. Various sperm parameters were tested after the sheep grew up, providing a theoretical basis for the effect of VA supplementation on the fertility of ruminants.

## Materials and methods

### Animals and treatments

A total of 32 hybrid F1 generation male lambs of Dorper sheep (♂) × Hu sheep (♀), which were newborns and had similar weights (3.5 ± 0.5 kg), were selected from a farm in Taigu county, Shanxi Province. All lambs were twins derived from the same father using artificial insemination technology. The lambs were randomly divided into the control and VA groups, with 16 in each group. They were reared with ewes and were free to drink their breast milk. The lambs were intramuscularly injected with corn oil (control group) or corn oil + 2500 IU/kg BW VA (PHR1235; Sigma, St. Louis, MO, USA) once a week at fixed times from birth to 3 weeks. The VA dose was selected based on our previous studies in mice (15) and cattle (16), which were converted to body weight. At 3 weeks of age, eight lambs from each group were randomly slaughtered to collect blood and testicular tissue. The remaining eight lambs in each group were weaned at 3 months of age, fed a backgrounding diet for 55 days, and then transferred to the finishing diet. From weaning to harvest, the daily intake of concentrate was increased from 0.3 to 1.75 kg, and grass hay was increased from 0.25 to 0.5 kg per lamb. The ingredients and nutrient composition of the diet, which contains 13,000 IU VA / kg concentrate, and the growth performance of the lambs have been previously published (17). They were slaughtered until 8 months of age for blood, testis, and epididymis tissue collection. Body and testis weights were measured to calculate the coefficients of the testes.

Testicular tissue from 3-week-old and 8-month-old sheep was used to obtain tissue slices, and the other was used to detect spermatogonial differentiation, cell cycle, steroidogenesis, antioxidation-related genes, RA, AMPK, AKT signaling, and the antioxidant enzyme levels. Blood was used to detect systemic steroid hormone. In addition, 8-month-old sheep epididymal sperm were collected to detect the expression of protein related to energy metabolism and sperm activity parameters.

### Hematoxylin and eosin staining

The testicular samples were fixed in 4% paraformaldehyde for 24 h, dehydrated in an ethanol gradient, and made transparent in xylene. The samples were then embedded in paraffin, and each sample was sectioned at a thickness of 6 μm using a microtome (Leica, Wetzlar, Germany). The sample sections were serially dewaxed, rehydrated with xylene and ethanol, and stained with hematoxylin and eosin. The stained sections were dehydrated, made transparent, and imaged under a microscope (Olympus, Tokyo, Japan).

### qRT-PCR analysis

The obtained testicular tissue was ground into a powder. Total RNA was extracted using RNAiso Plus (Takara, Tokyo, Japan) and reverse-transcribed into cDNA using the PrimeScript RT Reagent Kit with gDNA Eraser (Perfect Real Time) (Takara, Tokyo, Japan) according to the manufacturer's protocol. Primers were designed to span exons and were synthesized by Sangon Biotech Inc. (Shanghai, China). The primer sequences are shown in Table 1. qRT-PCR was conducted on a Bio-Rad Cfx system using the 2 × Real-time PCR Super mix (Mei5bio, Beijing, China). The procedure contained 38 cycles at 95°C for 15 s, 60°C for 15 s, and 72°C for 45 s. The amplification efficiency of all genes was calculated to ensure that they were in 90%−110%. The dissolution curves were unimodal. Ultimately, the relative abundance of mRNA expression was analyzed using the 2^−ΔΔCt^ method and normalized to *GAPDH*.

**Table 1:** Primer sequences for sheep qRT-PCR.

**Genes**	**Sequence (5' - 3')**	**Length (bp)**
*STRA8*	F:TATTTAGGGCCAAGCCAAGC	238
	R:ATTCAAAACTTGCCACTTTGAGG	
*KIT*	F:ACAAATCCATGCTCACACCCT	123
	R:ACTTCATACATGGGCTTCTGC	
*CYP11A1*	F:GCTTTGCCTTTGAGTCCATC	135
	R:TGAGCAGAGGGACACTGGTA	
*CYP17A1*	F:CACAATGAGAAGGAGTGGCA	83
	R:GGCGAGATGAGTTGTGTCCC	
*STAR*	F:CCACACTCTACGAGGAGATGC	152
	R:CTTCGGCAGCCATCCCTTGA	
*HSD3B1*	F:GGGAGGAATTTTCTAAGCTCCA	130
	R:TCAATGACAGAGGCGGTGTG	
*HSD17B3*	F:AACACGCCAGATGACTTCCA	154
	R:AGTACAGAGGCCAGGGAAAGA	
*RARA*	F:ATGACGCTGAGACTGGGTTG	134
	R:CCGTTTCCGCACATAGACCT	
*RARB*	F:CGGAGAGCTACGAGATGACG	71
	R:GGTTTCTTGGTGAGCCTTGC	
*RXRA*	F:CTCCCCATTTTCCACGACTC	132
	R:GGATAGCGGCGTGTACC	
*RORA*	F:TCAGCAACTACATCGACGGG	173
	R:GCAGTAGGGGAAGAAGCCTG	
*ADH1C*	F:TCGCTCTGGAAAGAGTGTCC	167
	R:TGGAAAGCTCCCATGTGCAA	
*ADH4*	F:AGAAAATGGGCACCAAGGGAA	80
	R:TTCATTGCTGAGGGGCTTGT	
*ALDH1A1*	F:CGCAACCGAGGAGAAACTCT	200
	R:TCATAGCCTCCATTGTCGCC	
*ALDH1A2*	F:AGCTCTGTGCTGTGGCAATA	101
	R:GTGGAAAGCCAGCCTCCTTG	
*CYP26A1*	F:TGACCCGCAATCTCTTCTCG	200
	R:CTCTCCTCTCTCCCACGAGT	
*CYP26B1*	F:CATCTCGTCCGTCTGTAGGTG	113
	R:TCGTAAGCCCCTTCCCTTGT	
*SOD2*	F:ATTGCTGGAAGCCCATCAAAC	193
	R:AGCAGGGGGATAAGACCTGT	
*CAT*	F:GCCTGTGTGAGAACATTGCG	121
	R:TCCAAAAGAGCCTGGATGCG	
*GPX4*	F:AAAGAGTTCGCTGCTGGCTA	143
	R:TTCCATTTGATGGCGTTTCCC	
*PCNA*	F:TCAAGTGGCGTGAACCTACA	143
	R:AGTATTTTGGACATGCTGGTGAG	
*CDK1*	F:TGCTCTTGACACAACACAGGA	151
	R:GCTTGGATCTGCTCTCGAAAA	
*CDK4*	F:ATCCCAATGTTGTCAGGCTC	264
	R:GCTTGACTGTCCCACCACTT	
*CCND1*	F:TCCTCTCCAAAATGCCGGTG	116
	R:CATGGAGGGTGGGTTGGAAA	
*CCND2*	F:AGTGCGTGCAGAAGGACATT	158
	R:ATGGGTCTTCGGAGTTGGGA	
*P21*	F:GAGCGATGGAACTTCGACTTTG	318
	R:GCGTTTGGAGTGGTAGAAATCTGT	
*P53*	F:GCCTCTCATCTCCGACTTCTTC	279
	R:CACGAAACCGAACGCTGCT	
*GAPDH*	F:TCGGAGTGAACGGATTTGGC	178
	R:CCGTTCTCTGCCTTGACTGT	

### Western blotting analysis

Western blotting was performed as previously described (18). Briefly, the tissues were powdered and added to an ice-cold protein extraction buffer. The obtained samples were centrifuged to obtain the supernatant, which was denatured. Target proteins were separated using sodium dodecyl-sulfate polyacrylamide gel electrophoresis (80 V, 30 min; 120 V, 1 h) and transferred onto a nitrocellulose membrane (100 V, 1.5 h). The membranes were then soaked in skim milk powder (1 h), primary antibody (4°C, overnight), or fluorescent secondary antibody (1 h). Protein bands were scanned using an infrared imaging system (LI-COR Biosciences, Lincoln, NE, US). Protein density was analyzed using ImageJ software (National Institutes of Health, Bethesda, MD, USA) and normalized to β-actin.

The antibodies against p-AMP-activated protein kinase (AMPK)α1 (4184S, 1:1000), protein kinase B (AKT; 9272S, 1:1000), p-AKT (4060S, 1:2000), initiation factor 4E binding protein 1 alpha (4EBP1α; 9644S, 1:1000) and p-4EBP1α (2855S, 1:1000) were purchased from the Cell Signaling Technology (Danvers, MA, USA). AMPKα1 (bs-10344R, 1:500) and β-actin (bsm-33036M, 1:8000) were obtained from the Biosynthesis Biotechnology Co., Ltd. (Beijing, China). Fluorescent secondary antibodies goat anti-rabbit IgG (926-32211, 1:20000) and goat anti-mouse IgG (926-68070, 1:20000) were obtained from LI-COR Biosciences (Lincoln, NE, USA).

### Enzyme-linked immunosorbent assay

The serum concentrations of testosterone (T), luteinizing hormone (LH), and follicle-stimulating hormone (FSH) were measured according to the manufacturer's instructions of the ELISA kits (MM-3460101, MM-3460401, and MM-124501, Jiangsu Meimian Industrial Co., Ltd, China). Briefly, serum was added to microtiter plate wells and incubated at 37°C for 30 min. Next, the horseradish peroxidase-conjugate reagent was added to the wells and incubated at 37°C for 30 min. The chromogen solution was added and incubated at 37°C for 10 min in the dark, and the reaction was stopped with a stop solution. The absorbance of each well was measured at 450 nm using a microplate reader (BioTek, Winooski, USA). The r^2^ (coefficient of regression) of fitted curves were all >0.996.

### Antioxidant analysis

Testicular tissue was powdered in liquid nitrogen, and 1 g of the powder was added to 9 mL of normal saline to obtain a 10% tissue homogenate. The supernatant was obtained by centrifuging at 800 g for 10 min at 4°C. The total protein concentration in the supernatant was measured using a BCA kit (Solarbio, Beijing, China) according to the manufacturer's instructions. Multiple enzyme activity was detected using biochemical kits, according to the manufacturer's protocol. Superoxide dismutase (SOD, A001-3), glutathione peroxidase (GSH-PX, A005-1), catalase (CAT, A007-1), malondialdehyde (MDA, A003-1), and total antioxidant capacity (T-AOC, A015-1) kits were purchased from Nanjing Jiancheng Bioengineering Institute (Nanjing, China). Their intra-assay coefficients of variation (CV) were 5.50%, 3.10%, 1.90%, 3.50% and 3.20%, respectively. And their inter-assay CV were 3.32%, 4.34%, 4.94%, 4.11% and 6.83%.

### Sperm evaluation

#### Computer-assisted sperm analysis

The right cauda epididymis of each sheep was cut into small pieces and soaked in 5 mL of sperm dilution solution containing 27 mg/mL Tris, 14 mg/mL citric acid, 10 mg/mL fructose, 100 IU/mL of penicillin, and 100 μg/mL of streptomycin, then it was gently shaken at 35°C for 30 min to let the sperm flow out freely. The sperm suspension was collected and placed onto preheated microscope slides. Sperm was observed under a phase contrast microscope (UB 200i Series, Olympus, Tokyo, Japan). Sperm motility, viability, density, average path velocity (VAP), straight-line velocity (VSL), and curvilinear velocity (VCL) were analyzed using the CASA system (ISAS^®^1.2, Proiser R&D, Valencia, Spain).

#### Sperm membrane integrity analysis

Sperm membrane integrity was assessed using a hypo-osmotic swelling test (HOST). First, 10 μL of a sperm suspension was added to 100 μL of a hypo-osmotic solution containing 13.5 mg/mL fructose and 7.35 mg/mL sodium citrate and incubated for 30 min at 35°C. Afterward, 10 μL of the mixture was evenly dripped onto microscope slides and air-dried. A total of 500 sperms were counted in randomly selected fields under a microscope (BX53, Olympus, Tokyo, Japan). The proportion of sperms with swollen and curved tails was calculated and regarded as the proportion of sperms with a completely functional membrane.

#### Sperm mitochondrial activity analysis

Sperm mitochondrial activity was assessed using 5,5',6,6'-tetrachloro-1,1',3,3'-tetraethylbenzimi-dazolylcarbocyanine iodide (JC-1) and propidium iodide (PI) double fluorescence staining. The sperm suspension was mixed with 1 mg/mL of JC-1 solution and 1 mg/mL of PI solution and incubated at 35°C for 30 min in the dark. Subsequently, Hancock's solution, containing 3.15 mg/mL of NaCl and 2.59% methanol, was added to the mixture to stop sperm movement. The samples were observed under a fluorescence microscope (BX53, Olympus, Tokyo, Japan), and 500 sperms were counted in multiple randomly selected fields. The yellow sperm tails indicated high mitochondrial membrane potential (MMP) and were regarded as having high mitochondrial activity.

#### Immunofluorescence

The sperm suspension was dripped evenly onto sticky glass slides and allowed to dry. The slides were immersed in 4% paraformaldehyde for 20 min, 1% Triton X-100 for 30 min, and 1% bovine serum albumin for 1 h. The primary antibody against CLUT3 (bs-1207R, 1:500, Bioss, Beijing, China) was added to the sperm surface at 4°C overnight. The next day, sperms were incubated with FITC-goat anti-rabbit IgG (BOSTER, Wuhan, China) for 1 h and DAPI solution (Sigma-Aldrich, St. Louis, MO, USA) for 20 min in the dark. Sperms were observed and photographed under a fluorescence microscope (BX53; Olympus, Tokyo, Japan).

### Statistical analysis

All experiments were repeated three times. The normal distribution and homogeneity of data variance were tested using the Shapiro-Wilk and Browne-Forsythe test, and significance analysis was conducted using a *t*-test in SPSS (version 22.0; IBM, Armonk, NY, USA). All values are expressed as the mean ± standard error of the mean (SEM). A *P* < 0.05 was considered statistically significant for all data.

## Results

### Effects of neonatal VA injection on spermatogenesis in sheep

**Figure 1:**
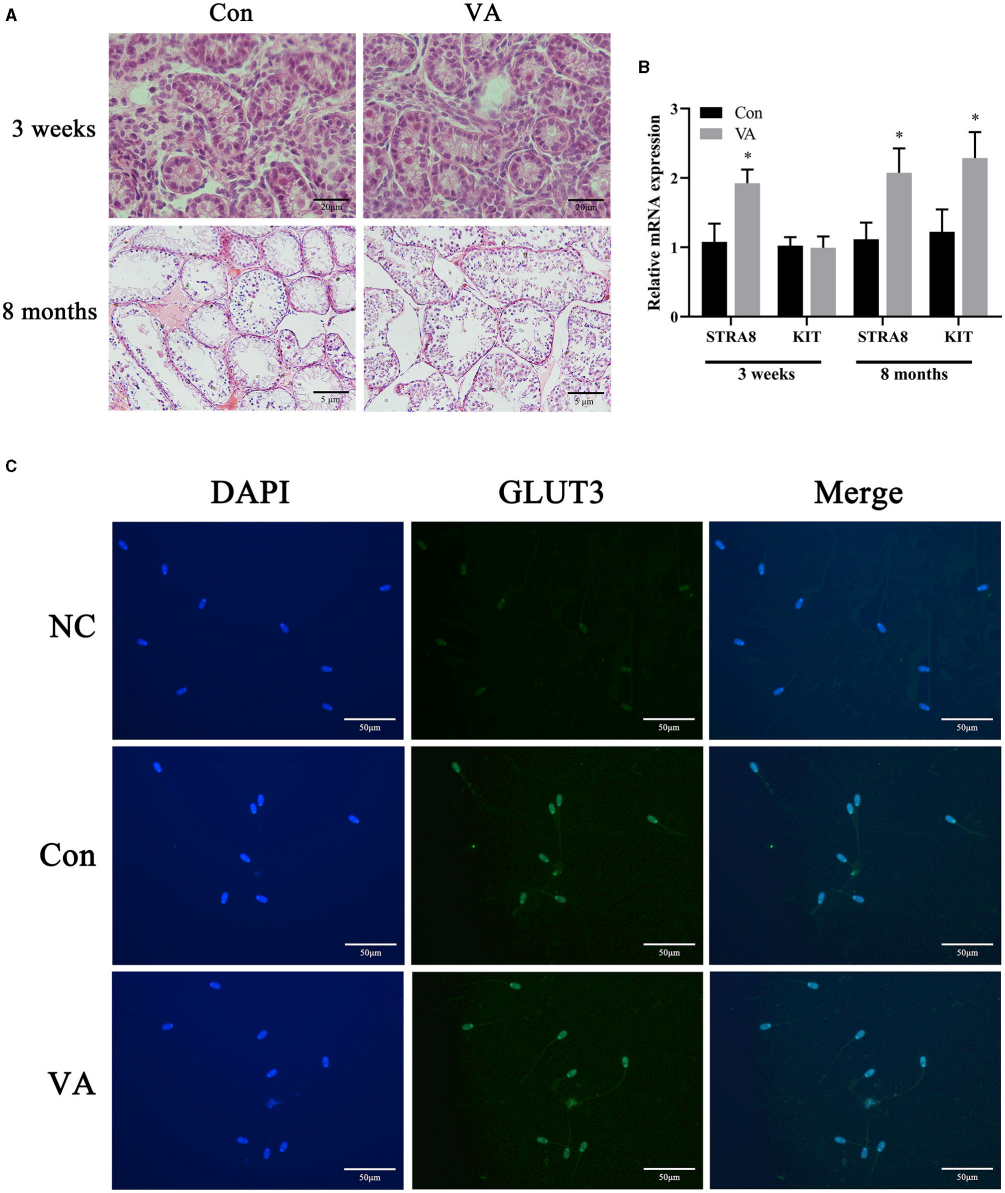
Spermatogonial differentiation and sperm energy metabolism related protein expression. HE staining of sheep testes **(A)**. Relative mRNA expression of spermatogonial differentiation related genes in sheep testes **(B)**. Immunofluorescence of sperm from 8-month-old sheep **(C)**. ^*^P < 0.05.

Through H&E staining, we observed that neonatal VA injection did not change the morphological structure of the testicular tissue or germ cells of 3-week-old sheep, but increased the diameter of seminiferous tubules and the number of spermatogonium and spermatocyte in 8-month-old sheep testis (Figure 1A). Neonatal VA injection promoted the mRNA expression of *STRA8* in the testes of 3-week-old sheep, and the mRNA expression of *STRA8* and KIT in the testes of 8-month -old sheep (*P* < 0.05) (Figure 1B). Immunofluorescence analysis showed that neonatal VA injection did not affect the expression of the energy metabolism-related protein glucose transporter 3 in the sperm of 8-month-old sheep (Figure 1C). Multiple sperm parameters were detected using the CASA, HOST, and JC-1/PI double fluorescence staining method (Table 2). Neonatal VA injection significantly increased sperm density (*P* < 0.05) but did not change sperm motility, viability, movement speed, membrane integrity, or MMP (*P* > 0.05) in the sperm suspension of 8-month-old sheep. In addition, testicular weight significantly increased in 8-month-old sheep in the VA group (*P* < 0.05), but there was no difference in the testicular coefficient (testicular weight/body weight) (*P* > 0.05).

**Table 2:** Testicular weight and sperm parameters of 8-month-old sheep.

**Parameter**	**Con**	**VA**
Weight of testis (kg)	0.18 ± 0.03^**b**^	0.28 ± 0.03^**a**^
Body weight (kg)	46.09 ± 2.11^**B**^	57.48 ± 2.69^**A**^
Coefficient of testis (%)	0.38 ± 0.06	0.51 ± 0.06
Motility (%)	45.60 ± 11.36	61.41 ± 8.21
Viability (%)	80.22 ± 7.10	88.60 ± 2.63
Density (million/mL)	68.09 ± 15.14^**b**^	129.84 ± 23.66^**a**^
VAP (μm/s)	39.65 ± 3.82	42.73 ± 3.45
VSL (μm/s)	28.39 ± 2.76	30.09 ± 2.50
VCL (μm/s)	69.43 ± 5.38	74.80 ± 4.18
HOST (%)	61.48 ± 2.77	65.71 ± 3.84
MMP (%)	60.56 ± 3.37	69.68 ± 4.64

All data were expressed as mean ± SEM. The ^a, b^different letters represent differences (*P* < 0.05) between different treatment groups. The ^A, B^different letters represent differences (*P* < 0.01) between different treatment groups. VAP, average path velocity; VSL, straight line velocity; VCL, curvilinear velocity; HOST, hypoosmotic swelling test; MMP, mitochondrial membrane potential.

### Effects of neonatal VA injection on testicular cell proliferation in sheep

**Figure 2:**
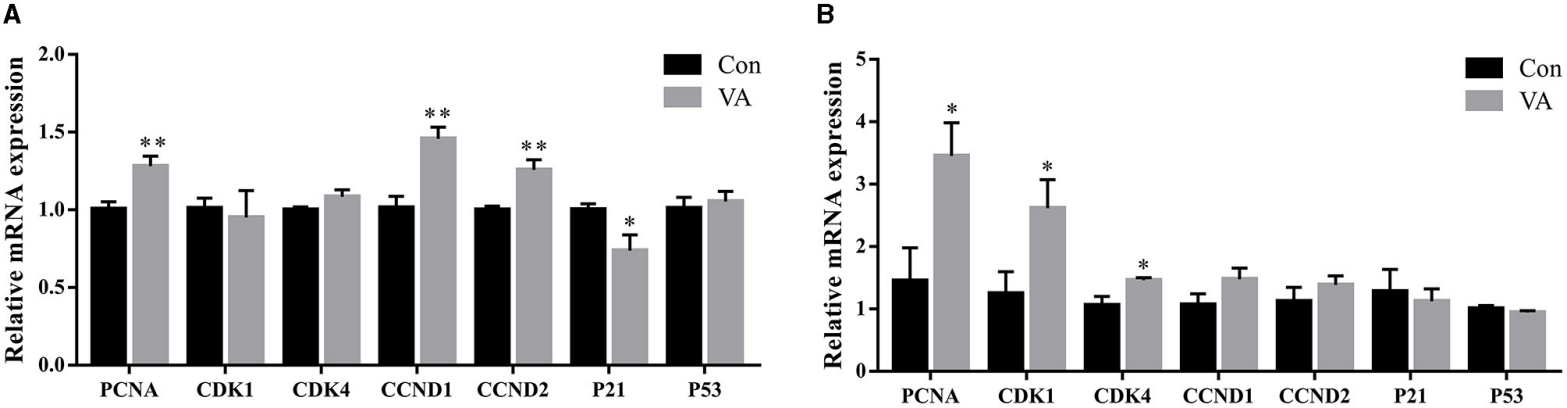
Proliferation of testicular cells. Relative mRNA expression of proliferative related genes in testes of 3-week-old sheep **(A)** and 8-month-old sheep **(B)**. *P < 0.05, **P < 0.01.

The mRNA expression of cell cycle-related genes in the testicular tissue was tested after neonatal VA injection. The results showed that the mRNA expression of *PCNA, CCND1*, and *CCND2* increased (*P* < 0.01), whereas the mRNA expression of *P21* decreased (*P* < 0.05) at 3 weeks of age. There was no effect on the mRNA expression of *CDK1, CDK4*, and *P53* at 3 weeks of age (Figure 2A). When the sheep grew to 8 months of age, the mRNA expression of *PCNA, CDK1*, and *CDK4* increased (*P* < 0.05), whereas the mRNA expression of *CCND1, CCND2*, and *P53* showed no difference in the testis (*P* > 0.05) compared with the control group (Figure 2B).

### Effects of neonatal VA injection on body hormone levels in sheep

**Figure 3:**
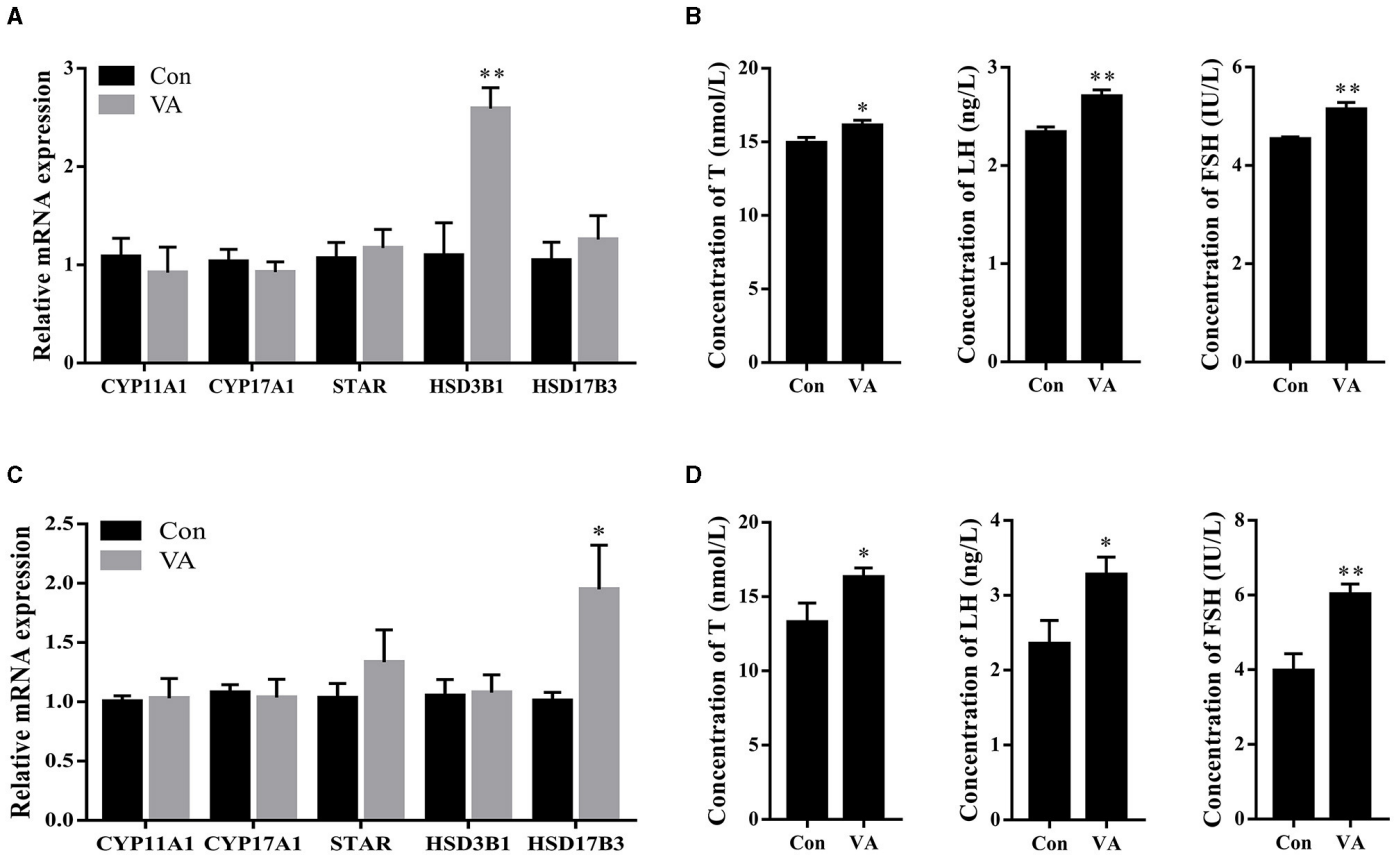
Synthesis and secretion of hormone. Expression of steroidogenesis related genes in testie of 3-week-old sheep **(A)**. Concentrations of hormone T, LH and FSH in serum of sheep at 3 weeks of age **(B)**. Expression of steroidogenesis related genes in testie of 8-month old sheep **(C)**. Concentrations of hormone T, LH and FSH in serum of sheep at 8 month of age **(D)**. *P < 0.05, **P < 0.01.

The expression of steroidogenesis-related genes in the testicular tissue and the concentrations of hormones in the serum were measured. The results showed that the mRNA expression of the steroidogenesis-related gene *HSD3B1* was upregulated (*P* < 0.01), whereas the mRNA expression of *CYP11A1, CYP17A1, STAR*, and *HSD17B3* remained unchanged (*P* > 0.05) in the testes of 3-week-old sheep after neonatal VA injection (Figure 3A). The mRNA expression of *HSD17B3* increased (*P* < 0.05), whereas there was no difference in the mRNA expression of *CYP11A1, CYP17A1, STAR*, or *HSD3B1* (*P* > 0.05) in the testes of 8-month-old sheep after neonatal VA injection (Figure 3C). The levels of serum hormones T, LH, and FSH in the VA group at 3 weeks and 8 months of age were higher than those in the control group using ELISA (*P* < 0.05) (Figures 3B, D).

### Effects of neonatal VA injection on antioxidant status in sheep testis

**Figure 4:**
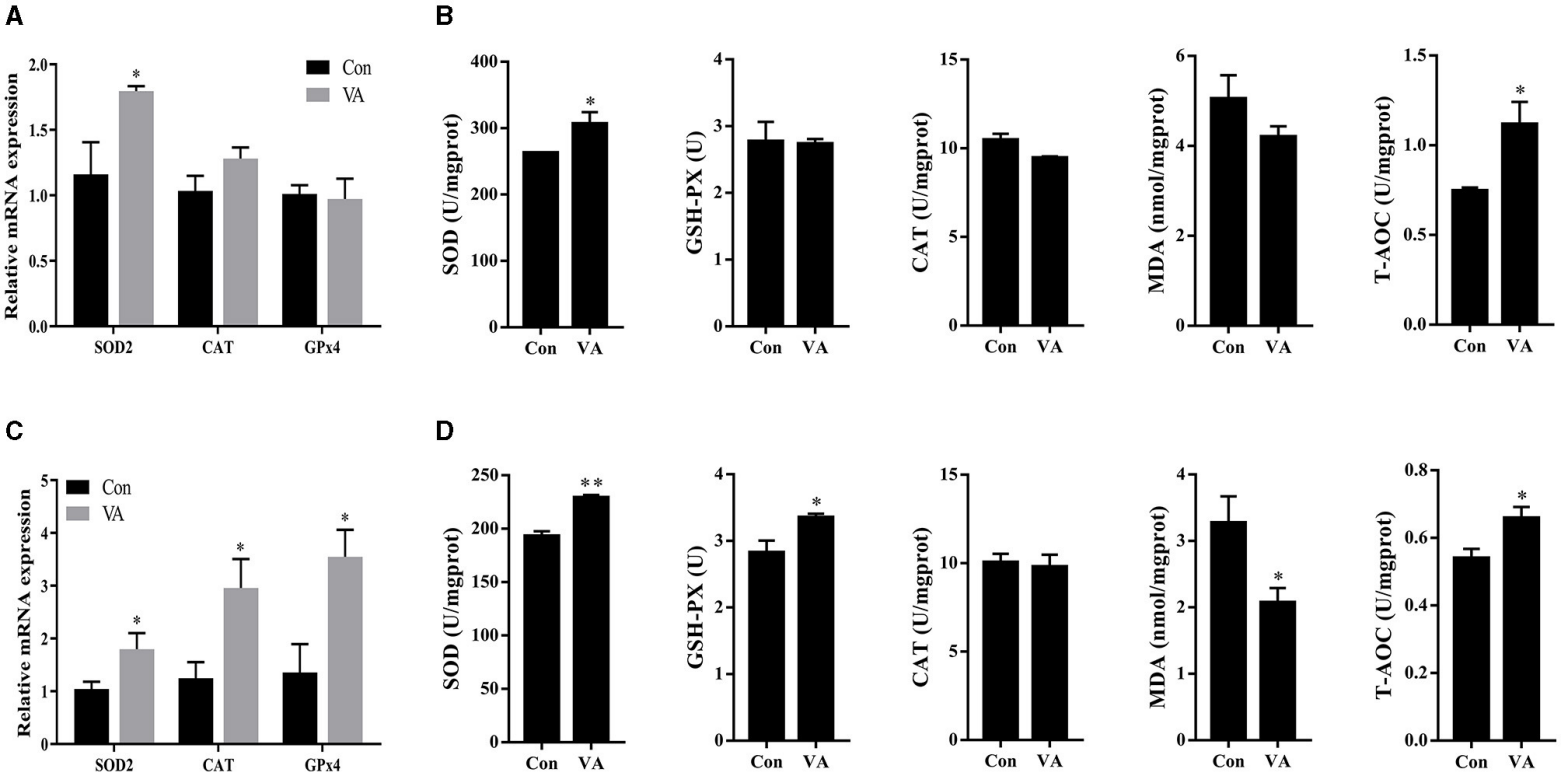
Antioxidant status of testis. Relative mRNA expression of antioxidant related genes in testes of 3-week-old sheep **(A)**. Abundance of antioxidant enzymes in testes of 3-week-old sheep **(B)**. Relative mRNA expression of antioxidant related genes in testes of 8-month-old sheep **(C)**. Abundance of antioxidant enzymes in testes of 8-month-old sheep **(D)**. *P < 0.05, **P < 0.01.

The abundance of antioxidant-related genes and antioxidant enzymes in sheep testes was detected. The results showed that the mRNA expression of *SOD2* increased, whereas the mRNA expression of *CAT* and *GPx4* showed no difference in the testes at 3 weeks of age after neonatal VA injection (*P* < 0.05) (Figure 4A). The abundance of the antioxidant enzymes SOD and T-AOC increased (*P* < 0.05), whereas the abundances of GSH-PX, CAT, and MDA were not affected (*P* > 0.05) in the testes of 3-week-old sheep (Figure 4B). Similarly, the mRNA expression levels of *SOD2, CAT*, and *GPx4* increased (*P* < 0.05) in the testes of 8-month-old sheep in the VA group (Figure 4C). The levels of the antioxidant enzymes SOD, GSH-PX, and T-AOC increased (*P* < 0.05), MDA decreased (*P* < 0.05), and CAT remained unchanged in the testes at 8 months of age (*P* > 0.05) (Figure 4D).

### Effects of neonatal VA injection on RA signaling pathway in sheep testis

**Figure 5:**
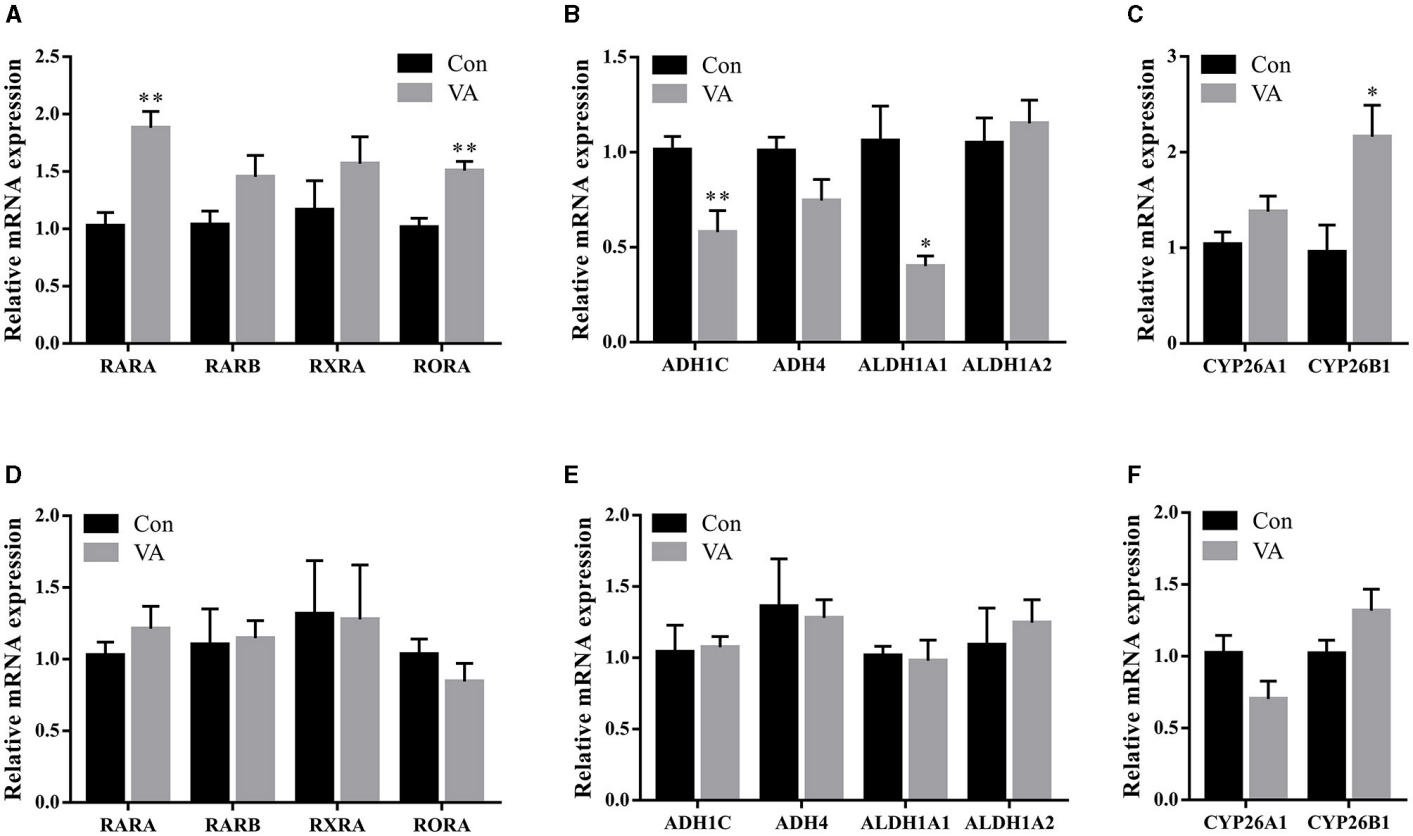
Expression of key genes in RA signaling pathway. Relative mRNA expression of RA receptor **(A)**, RA synthetase **(B)**, RA lyase **(C)** in testes of 3-week-old sheep. Relative mRNA expression of RA receptor **(D)**, RA synthetase **(E)**, RA lyase **(F)** in testes of 8-month-old sheep. *P < 0.05, **P < 0.01.

RA is an intermediate VA metabolite. To explore the regulation of VA in the sheep testes, we measured the expression of genes related to the RA signaling pathway. After neonatal VA injection, the mRNA expression of the RA receptors RARA and RAR-related orphan receptor A was upregulated (*P* < 0.01), whereas the mRNA expression of the RA receptor RARB and RXRA did not differ in the testes of 3-week-old sheep (*P* > 0.05) (Figure 5A). Moreover, the mRNA expression of RA synthetases *ADH1C* (*P* < 0.01) and *ALDH1A1* (*P* < 0.05) was downregulated, whereas the mRNA expression of RA synthetases *ADH4* and *ALDH1A2* was not affected (*P* > 0.05) (Figure 5B). The mRNA expression of the RA lyase *CYP26B1* was upregulated (*P* < 0.05), whereas the mRNA expression of the RA lyase *CYP26A1* was not affected (*P* > 0.05) (Figure 5C). However, there were no significant differences in RA receptor, RA synthetase, or RA lyase expression in the testes of 8-month-old sheep between the two groups (*P* > 0.05) (Figures 5D–F).

### Effects of neonatal VA injection on AMPK/AKT signaling pathway in sheep testis

**Figure 6:**
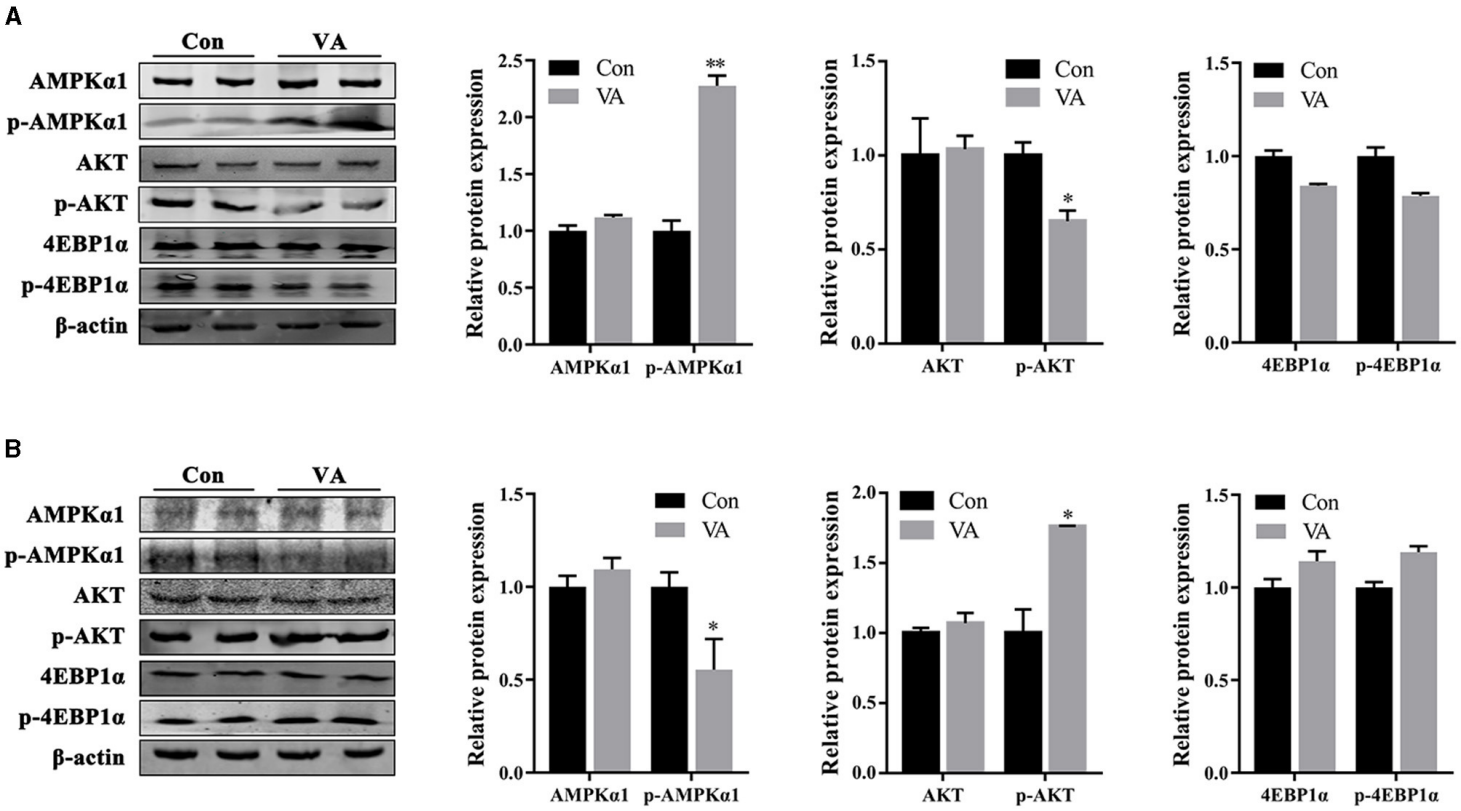
Protein abundance of AMPK and AKT signaling pathways. Western blot were performed to determine the activity of signaling pathway in testes of 3-week-old sheep **(A)** and 8-month-old sheep **(B)**. *P < 0.05, **P < 0.01.

The protein abundances of AMPK and AKT signaling pathways in the testes of 3-week-old and 8-month-old sheep were measured after neonatal VA injection in neonates. The results showed no difference in the total protein abundance of AMPK, AKT, and 4EBP1α in the testes at 3 weeks or 8 months of age (Figures 6A, B). The abundance of phosphorylated protein p-AMPK increased (*P* < 0.01), p-AKT decreased (*P* < 0.05), and p-4EBP1α remained (*P* > 0.05) at 3 weeks of age (Figure 6A). The abundance of phosphorylated protein p-AMPK decreased (*P* < 0.05), p-AKT increased (*P* < 0.05), and p-4EBP1α was not affected (*P* > 0.05) at 8 months of age (Figure 6B).

## Discussion

In male mammals, germ cells in the testes are isolated from RA signaling, maintain mitotic activity, and stop at the G0/G1 phase of mitosis during the fetal stage. After birth, the developmental transitions of spermatogenesis include germ cell proliferation, transition to spermatogonia, spermatogonia differentiation, and initiation of primary spermatocyte meiosis, all of which are strictly regulated by RA (19). RA induces the expression of *STRA8* and *KIT* in undifferentiated rodent spermatogonocytes (20–22). *KIT* is a marker of spermatogonial differentiation. *STRA8* is a marker gene for meiosis initiation that promotes spermatogonial differentiation (23). Although neonatal VA supplementation did not cause morphological changes in the testes of lambs, the expression of *STRA8* in the testis was increased. Neonatal VA supplementation may promote spermatogonial differentiation and primary spermatocyte meiosis in lamb testes. Compared with controls, testis weight, seminiferous tubule diameter, number of spermatogonium and spermatocyte, and sperm density increased significantly when VA-supplemented lambs grew, suggesting that neonatal VA supplementation promote the proliferation of testicular cells to improve the spermatogenic potential of sheep throughout their life. In addition, motility, viability, movement speed, membrane integrity, MMP, and expression of the energy metabolism-related protein glucose transporter 3 in sperm indicated that neonatal VA supplementation did not improve sperm activity in adult sheep.

The differentiation ability of spermatogonocytes is closely associated with their proliferative activity. To determine whether neonatal VA supplementation affected the proliferative ability of testicular cells, cell proliferation-related genes were examined. As a marker gene for cell proliferation, *PCNA* regulates the formation of chromatin, DNA damage repair, and separation of sister chromatids, enabling rapid and accurate replication of genomic DNA (24). The cell cycle-related genes *CDK1, CDK4, CCND1*, and *CCND2* induce mitosis (25). CCNDs bind to CDKs to form complexes that promote the cell cycle from G1 to S phase (26, 27). P21 and P53 are cell cycle inhibitors that lead to cell cycle arrest in response to various stimuli by inhibiting retinoblastoma protein phosphorylation and E2F transcription factor release (28–30). The results revealed that neonatal VA supplementation increased the proliferation of testicular cells and promoted the growth of the testes by synergistically regulating *PCNA, CCND1, CCND2*, and *P21* in lamb testes, thus enlarging the testes after sheep growth. Higher expression of *PCNA, CDK1*, and *CDK4* in the testes of adult sheep may be associated with stronger spermatogenic ability.

Hormones regulate spermatogenesis in the testes. Higher levels of FSH, LH, and T were detected in the serum of both lambs and adults following neonatal VA supplementation. FSH secreted by the pituitary gland is transported to the testes and directly promotes the proliferation of Sertoli cells. LH secreted by the pituitary gland stimulates Leydig cells to secrete T, which binds to the androgen receptor on Sertoli cells to further regulate Sertoli cell proliferation (2, 31). Sertoli cells provide nutritional support in seminiferous tubules and spontaneously synthesize endogenous RA, which regulates the expression of STRA8 and meiosis in spermatocytes through paracrine signals (8). FSH, LH, and T indirectly promote spermatocyte meiosis by acting on Sertoli or Leydig cells. The higher levels of gonadotropin and T suggested that neonatal VA supplementation led to a higher long-term meiotic capacity of ovine testis spermatocytes. Cholesterol, a substrate for T biosynthesis, is converted to pregnenolone by CYP11A1, which is subsequently converted to progesterone by HSD3B1. Progesterone is converted to androstenadione by CYP17A1, and T is synthesized from androstenedione by HSD17B3. STAR is an intracellular cholesterol carrier (32). The expression of *HSD3B1* in lamb testes and *HSD17B3* in adult sheep testes was upregulated by neonatal VA supplementation, which was consistent with the increased T levels. However, *in vitro* studies have reported that VA and RA regulate T synthesis by promoting the expression of STAR and CYP17A1 in Leydig cells. Specific blocking of RA signaling in mouse Leydig cells results in decreased expression of CYP17A1 and T levels, and VA deficiency in rats results in decreased expression of HSD3B1 and CYP11A1 (33, 34). Therefore, the regulation of steroidogenesis-related genes by RA may differ between sheep and rodent testis Leydig cells.

Vitamins may protect the sperm against oxidative stress and affect male fertility (35, 36). Serum VA levels are significantly decreased in men with infertility, and VA reduces sperm DNA breakage and lipid peroxidation through its antioxidant action (37). To further investigate whether the improvement in the reproductive ability of sheep by neonatal VA supplementation is related to the level of oxidative stress in the testes, we measured the antioxidant enzymes in the testes and found that neonatal VA supplementation increased the concentration of antioxidant enzymes and total antioxidant levels in both lamb and adult sheep testes. SOD catalyzes the conversion of superoxide free radicals to hydrogen peroxide, and loss of SOD activity is related to male sterility (38). GSH-Px and CAT break down hydrogen peroxide, protecting cells from oxidative damage caused by peroxides and maintaining cell membrane integrity (39). MDA is the final product of lipid peroxidation; therefore, changes in the MDA concentration can be used as a sign of oxidative stress and reflect the degree of cell damage (40). In addition, the increased expression of antioxidant-related genes confirmed the improved antioxidant capacity.

VA regulates downstream functional genes through the RA signaling pathway after diffusion into the cells. The RA signaling pathway is composed of the RA receptors (RAR, RXR), RA synthetases (ADH, ALDH), and RA lyase (CYP26). Periodic changes in RA levels in the seminiferous tubules of adult mammals are co-regulated by ADH and ALDH, which catalyze RA synthesis, and CYP26, which degrades RA. Periodic RA signals induce meiosis initiation to ensure that sperms are produced at a constant rate throughout reproductive life (19). ALDH1A knockout in mouse Sertoli cells prevents local RA production and blocks spermatogonal differentiation and meiosis initiation (41). CYP26B1 forms a catabolic barrier in Sertoli cells by degrading RA, preventing RA outside the spermatogenic tubules from reaching germ cells, and maintaining stable endogenous RA levels. CYP26B1 knockout in Sertoli cells increased endogenous RA in spermatogenic tubules and meiosis-related gene expression (42). The RA receptor RARA was upregulated in the testes of lambs after neonatal VA supplementation, suggesting that RA signaling in the testis was activated. The mRNA expression of the RAR-related orphan receptor A was also upregulated. ROR is involved in T biosynthesis and the regulation of semen quality and male fertility (43, 44). RAR activates its transcription (45). In addition, decreased RA synthetase and increased RA lyase levels indicated that the RA concentration was also elevated on the lateral side of the spermatogenic tubules. RA synthetase and lyase function together as the BTB. However, there was no difference in RA signaling between the control and VA groups after the sheep grew. Neonatal VA supplementation did not continuously activate the RA signaling pathway for a long time.

The AMPK and AKT signaling pathways have been proven to be two extremely important regulatory pathways in testicular development and spermatogenesis (46). AKT positively regulates the downstream proteins, mTOR, 4EBP1, and p70S6K, whereas AMPK inhibits their activities (47). AMPK participates in the regulation of tight junctions in testis cells (48). Tight junctions between the Sertoli cells constitute the BTB, providing a suitable microenvironment for spermatogenesis. Inhibition of AMPK signaling in Sertoli cells leads to the destruction of tight junctions between Sertoli and germ cells, the shedding of germ cells, and a decline in the fertility of mice (49). AKT signaling disrupts BTB structure by regulating protein synthesis and the cytoskeleton in Sertoli cells (50–52). Neonatal VA supplementation stimulates the activity of the AMPK signaling pathway and inhibits the activity of the AKT signaling pathway in the testis. It has been speculated that AMPK and AKT jointly enhance the junctions between Sertoli cells to form a stronger BTB and maintain periodic RA levels in the spermatogenic tubules. This result is consistent with the changes in the expression of RA synthetase and lyase, the key proteins that constitute the BTB. In addition to regulating intercellular junctions, AMPK inhibits the proliferation of Sertoli cells by mediating G1/S phase cell cycle arrest (53, 54). AKT also promotes the proliferation and differentiation of spermatogonia and Sertoli cells in the testes (50, 55). RA signaling initiated efficient mRNA translation associated with spermatogonal differentiation via the AKT signaling pathway (56). The phosphorylation of AMPK decreased, and that of AKT increased in the testes of adult sheep in the VA group, suggesting that these two signaling pathways synergistically regulate the maintenance of high spermatogenic ability in adult sheep. There was no significant difference in the expression of the downstream protein p-4EBP1 in the VA group in either the lamb or adult sheep testes; however, this slight trend was consistent with the regulatory effects of p-AMPK and p-AKT. Therefore, we speculate that changes in AMPK and AKT signaling pathway activity caused by neonatal VA supplementation may play different regulatory roles in the testes of lambs and adult sheep.

## Conclusions

In conclusion, neonatal VA supplementation promotes testis development and improves spermatogenesis by regulating spermatogonial differentiation, spermatocyte meiosis, cell proliferation, hormone levels, and antioxidant levels in sheep testes. VA activates the RA signaling pathway and enhances BTB and spermatogenesis by regulating the AMPK and AKT signaling pathways in sheep testes. The rapid replenishment and renewal of spermatogenic cells in the testis of adult sheep promote the continuous production and release of sperm, and the increase in serum testosterone levels also stimulates male sexual behavior. The higher semen density and libido of rams are conducive to improving the mating rate in the actual breeding process. Therefore, VA supplementation may improve the reproductive potential of male lambs.

## Data availability statement

The original contributions presented in the study are included in the article/Supplementary material, further inquiries can be directed to the corresponding author.

## Ethics statement

The animal studies were approved by the Animal Protection and Utilization Committee of Shanxi Agricultural University. The studies were conducted in accordance with the local legislation and institutional requirements. Written informed consent was obtained from the owners for the participation of their animals in this study.

## Author contributions

YL: Conceptualization, Data curation, Investigation, Methodology, Writing – original draft. PS: Conceptualization, Methodology, Writing – original draft. JiZ: Methodology, Writing – original draft. WZ: Methodology, Writing – original draft. XLi: Conceptualization, Methodology, Writing – original draft. XLv: Conceptualization, Funding acquisition, Writing – original draft. JuZ: Funding acquisition, Resources, Supervision, Writing – review & editing.

## Supplementary material

Supplementary Data Sheet 1 Original images of gels.

Supplementary Data Sheet 2 Microscopy images.
