# Supplementary figures and images for: Neonatal vitamin A supplementation improves sheep fertility potential
Source: Front Vet Sci. 2024 May 2;11:1370576. doi: 10.3389/fvets.2024.1370576 (PMC11097686; doi:10.3389/fvets.2024.1370576)

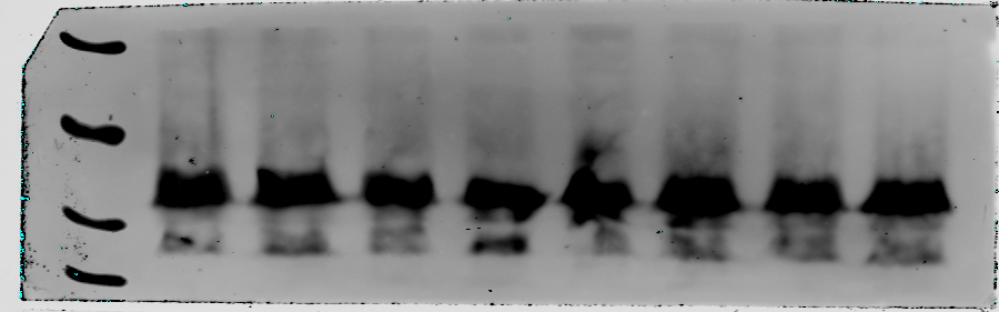

Supplement: Supplementary Data Sheet 1 — Original images of gels. [file Data_Sheet_1.zip › Original images of gels/3-week-old/4EBP1α.tif]

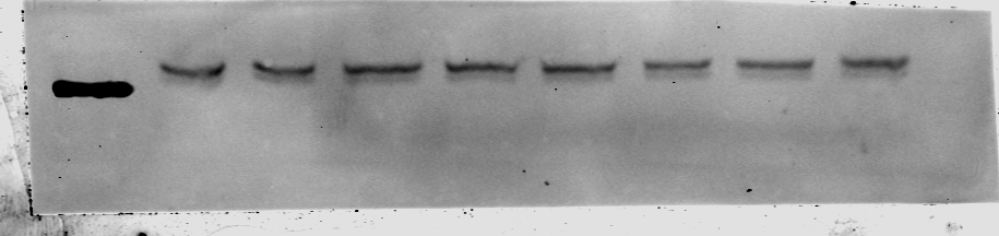

Supplement: Supplementary Data Sheet 1 — Original images of gels. [file Data_Sheet_1.zip › Original images of gels/3-week-old/AKT.tif]

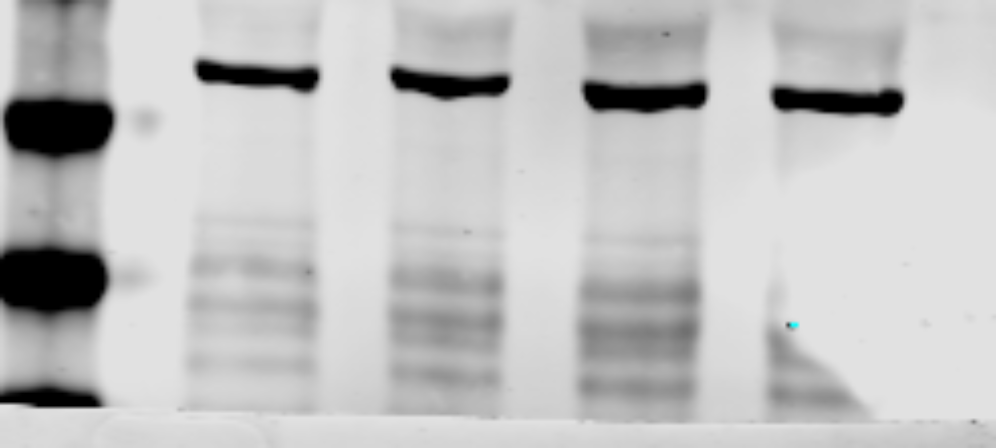

Supplement: Supplementary Data Sheet 1 — Original images of gels. [file Data_Sheet_1.zip › Original images of gels/3-week-old/AMPKα1.tif.tif.tif]

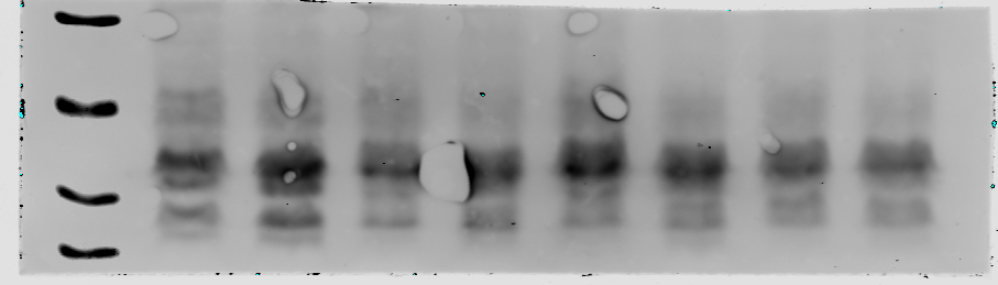

Supplement: Supplementary Data Sheet 1 — Original images of gels. [file Data_Sheet_1.zip › Original images of gels/3-week-old/p-4EBP1α.tif]

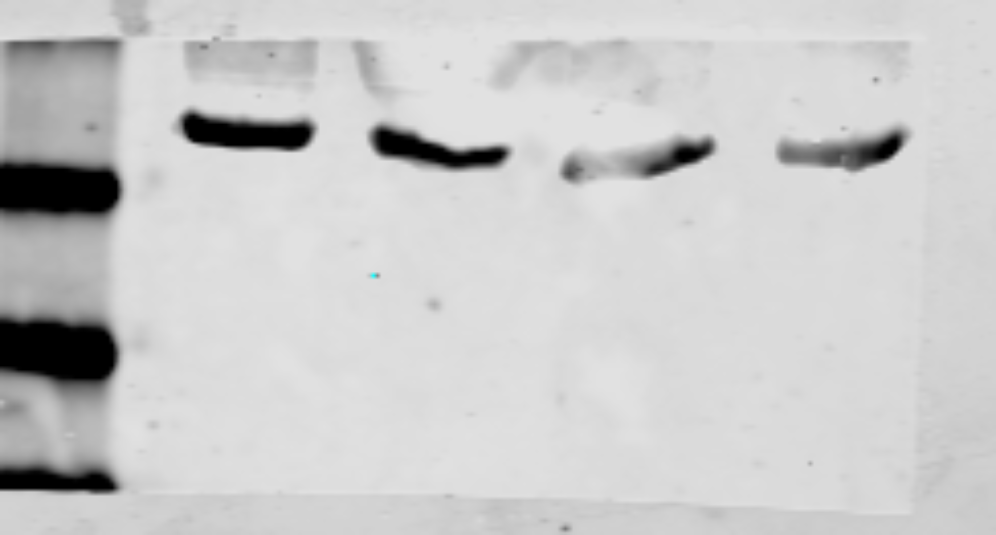

Supplement: Supplementary Data Sheet 1 — Original images of gels. [file Data_Sheet_1.zip › Original images of gels/3-week-old/p-AKT.tif.tif]

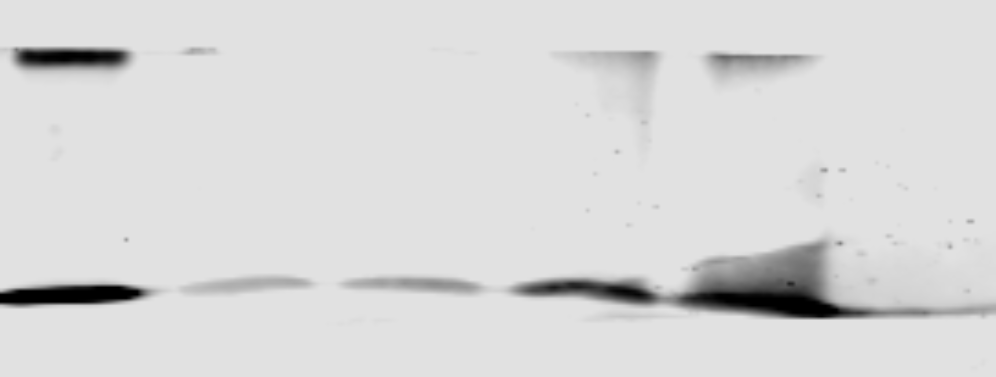

Supplement: Supplementary Data Sheet 1 — Original images of gels. [file Data_Sheet_1.zip › Original images of gels/3-week-old/p-AMPKα1.tif.tif]

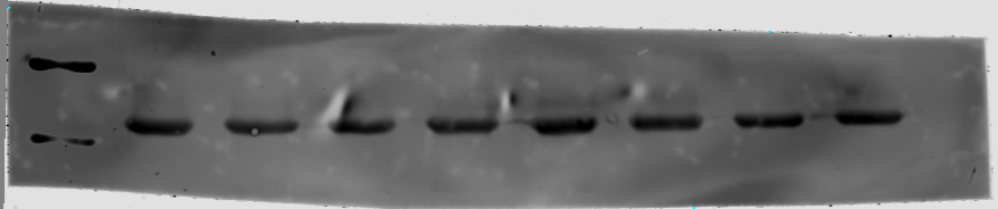

Supplement: Supplementary Data Sheet 1 — Original images of gels. [file Data_Sheet_1.zip › Original images of gels/3-week-old/β-actin.tif]

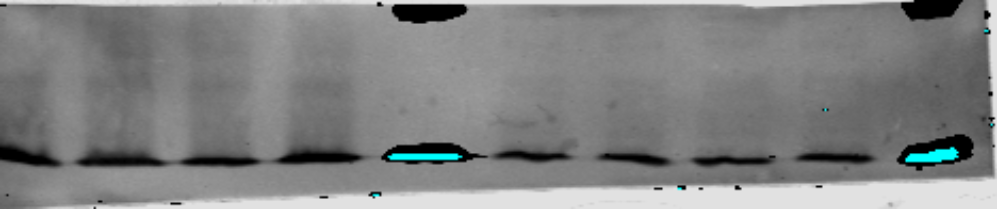

Supplement: Supplementary Data Sheet 1 — Original images of gels. [file Data_Sheet_1.zip › Original images of gels/8-month-old/4EBP1α.tif.tif]

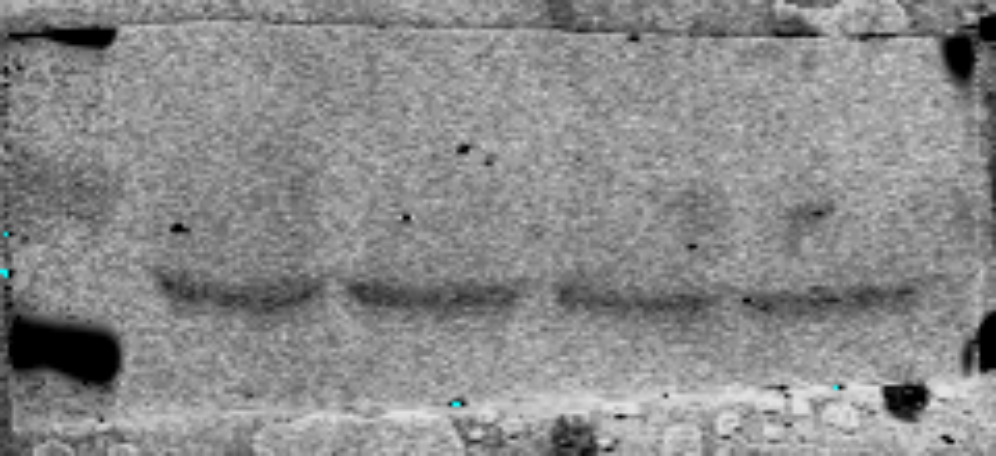

Supplement: Supplementary Data Sheet 1 — Original images of gels. [file Data_Sheet_1.zip › Original images of gels/8-month-old/AKT.tif.tif]

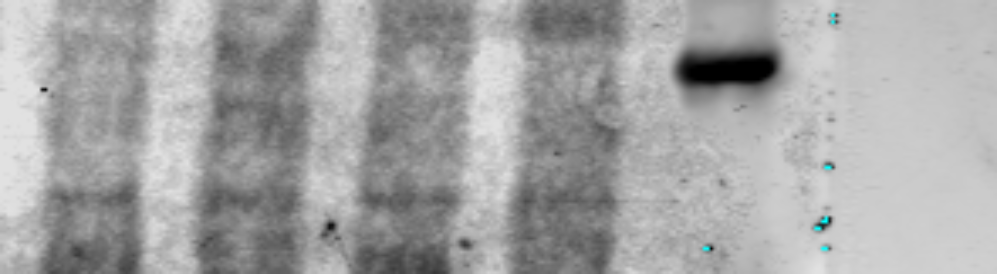

Supplement: Supplementary Data Sheet 1 — Original images of gels. [file Data_Sheet_1.zip › Original images of gels/8-month-old/AMPKα1.tif.tif]

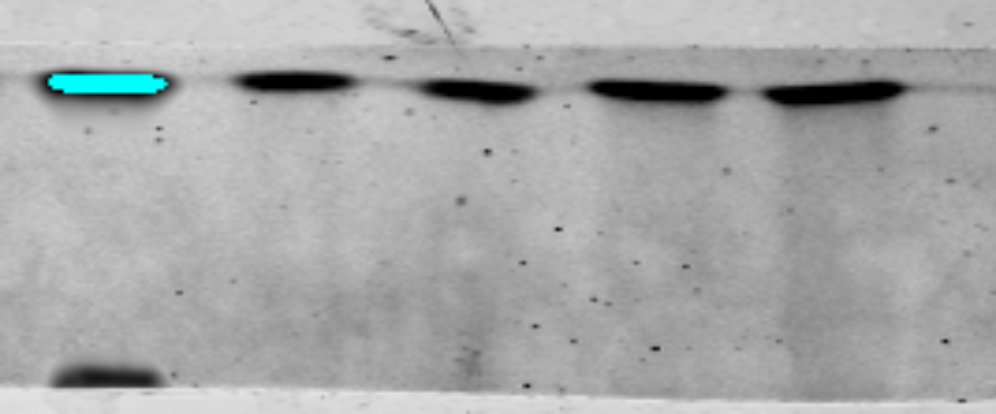

Supplement: Supplementary Data Sheet 1 — Original images of gels. [file Data_Sheet_1.zip › Original images of gels/8-month-old/p-4EBP1α.tif.tif.tif]

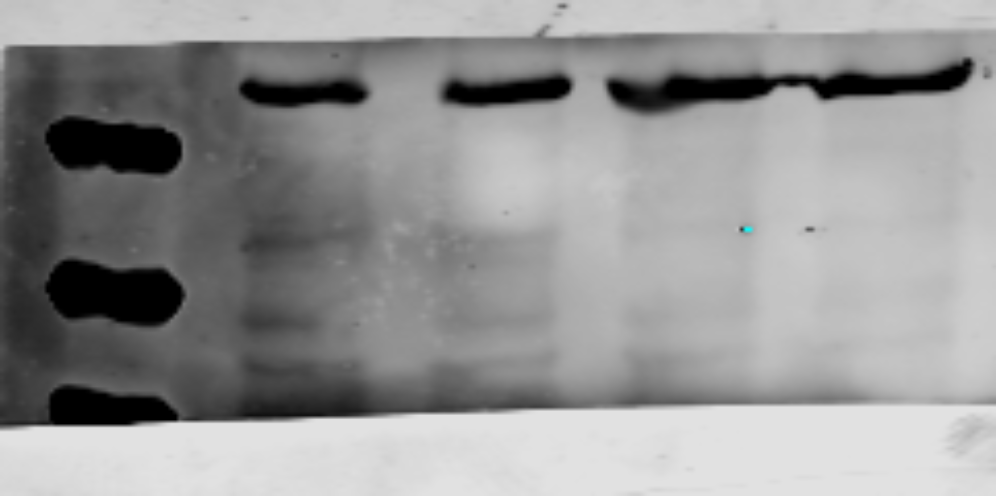

Supplement: Supplementary Data Sheet 1 — Original images of gels. [file Data_Sheet_1.zip › Original images of gels/8-month-old/p-AKT.tif.tif]

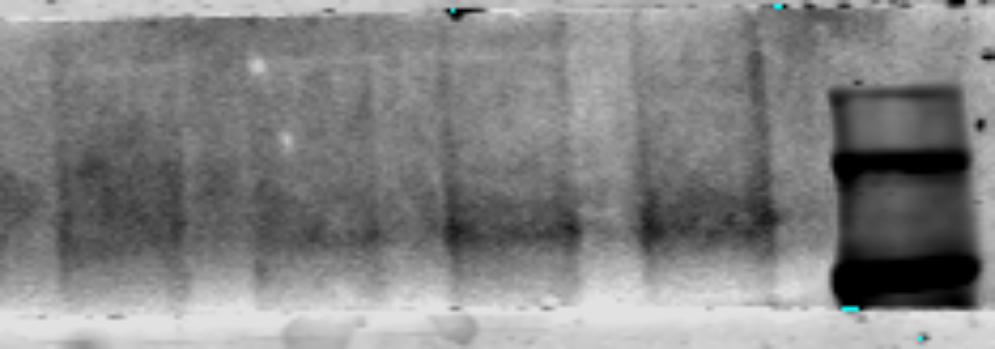

Supplement: Supplementary Data Sheet 1 — Original images of gels. [file Data_Sheet_1.zip › Original images of gels/8-month-old/p-AMPKα1.tif.tif]

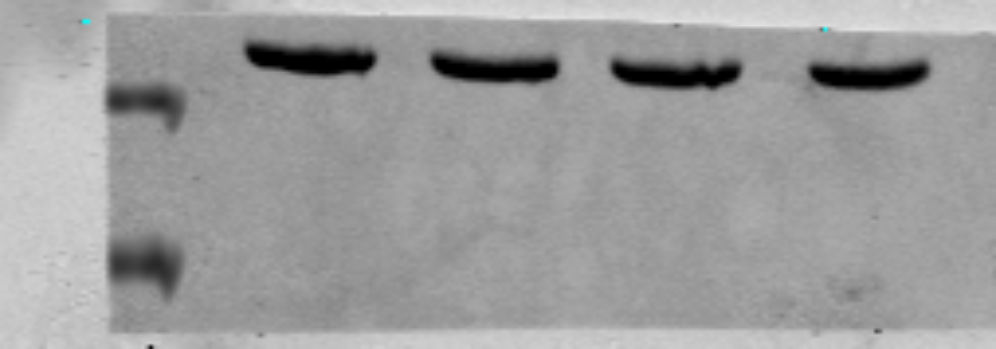

Supplement: Supplementary Data Sheet 1 — Original images of gels. [file Data_Sheet_1.zip › Original images of gels/8-month-old/β-actin.tif]

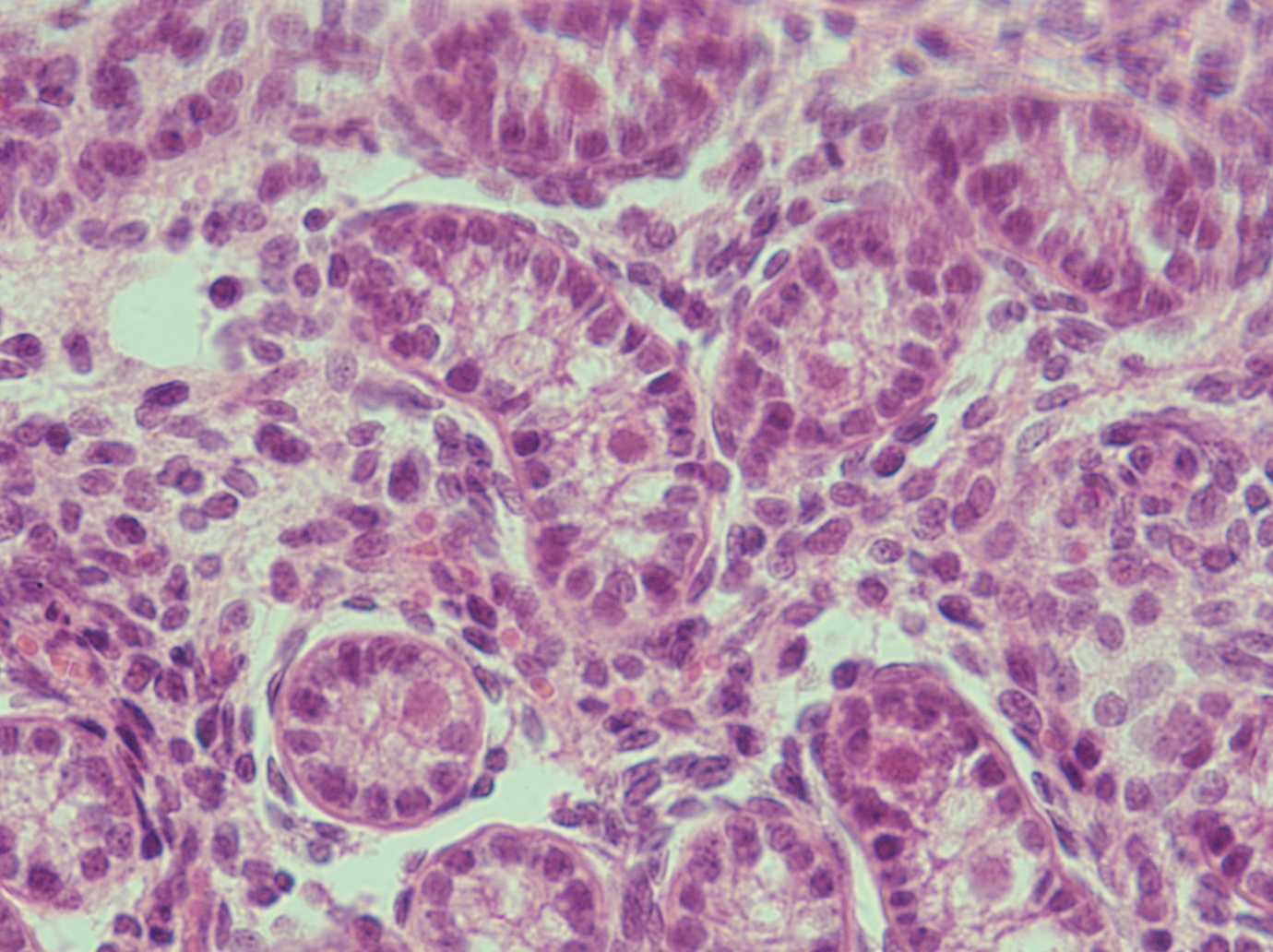

Supplement: Supplementary Data Sheet 2 — Microscopy images. [file Data_Sheet_2.zip › Microscopy images/HE staining/3 weeks Con.tif]

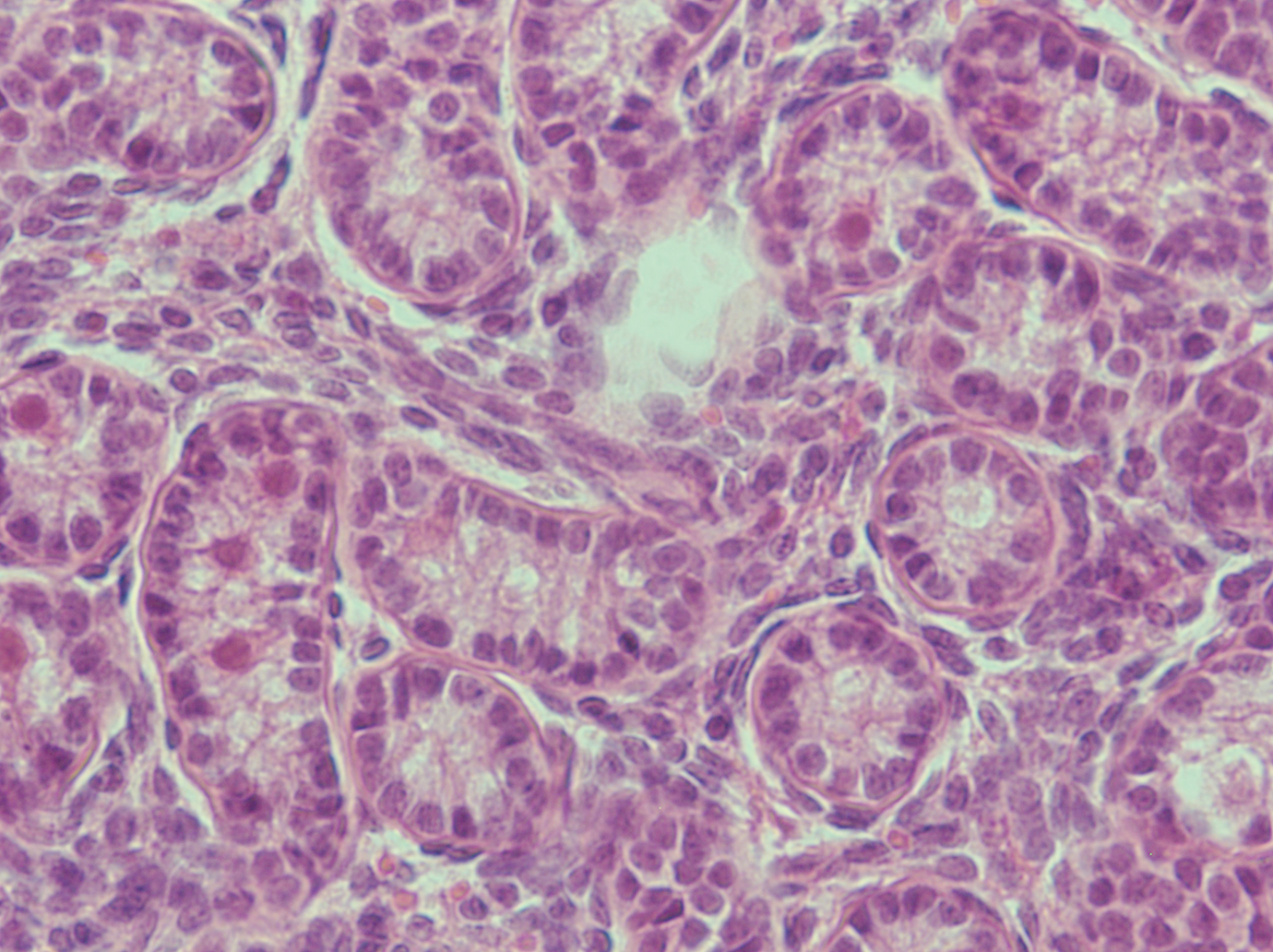

Supplement: Supplementary Data Sheet 2 — Microscopy images. [file Data_Sheet_2.zip › Microscopy images/HE staining/3 weeks VA.tif]

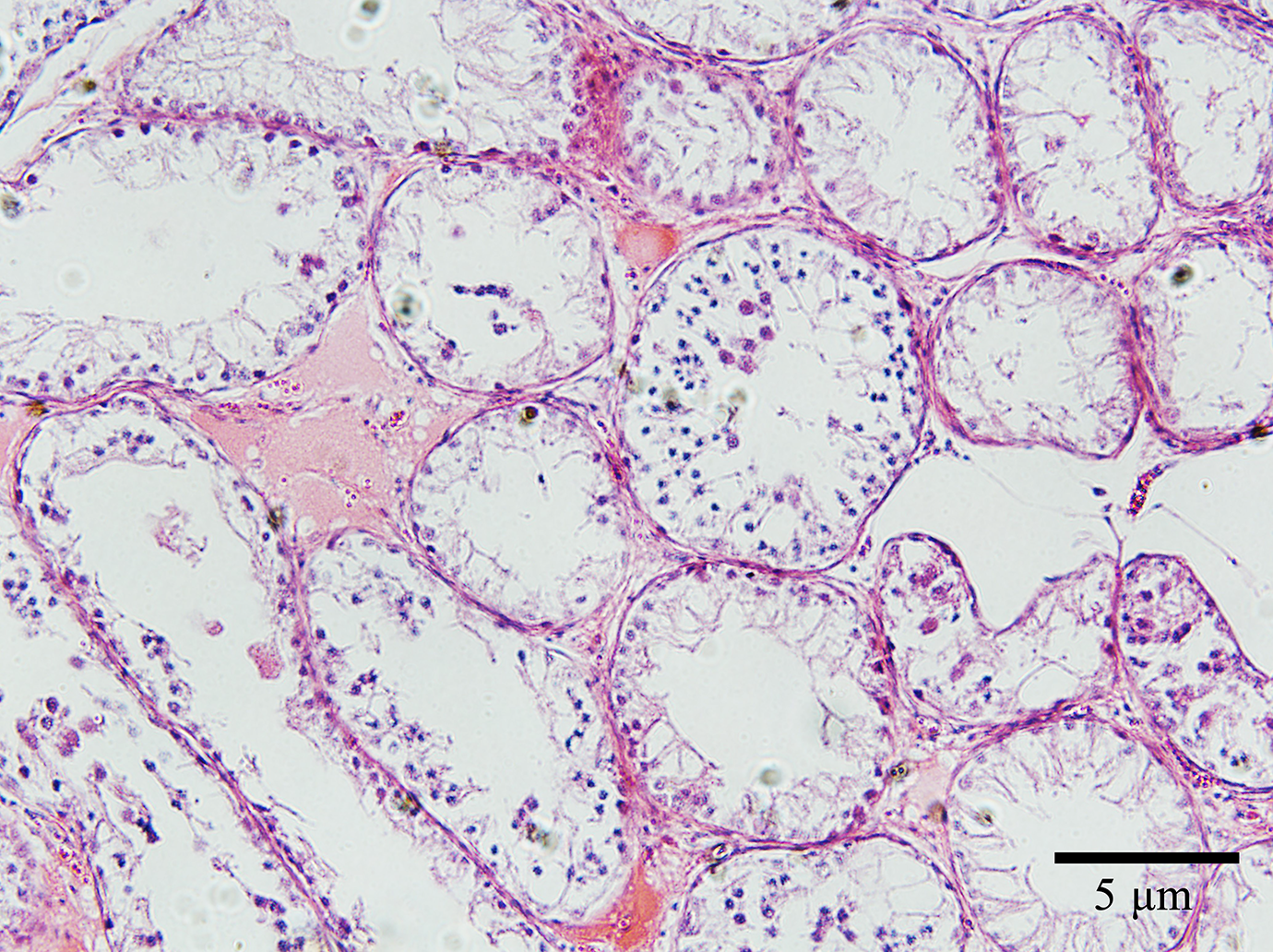

Supplement: Supplementary Data Sheet 2 — Microscopy images. [file Data_Sheet_2.zip › Microscopy images/HE staining/8 months Con.tif]

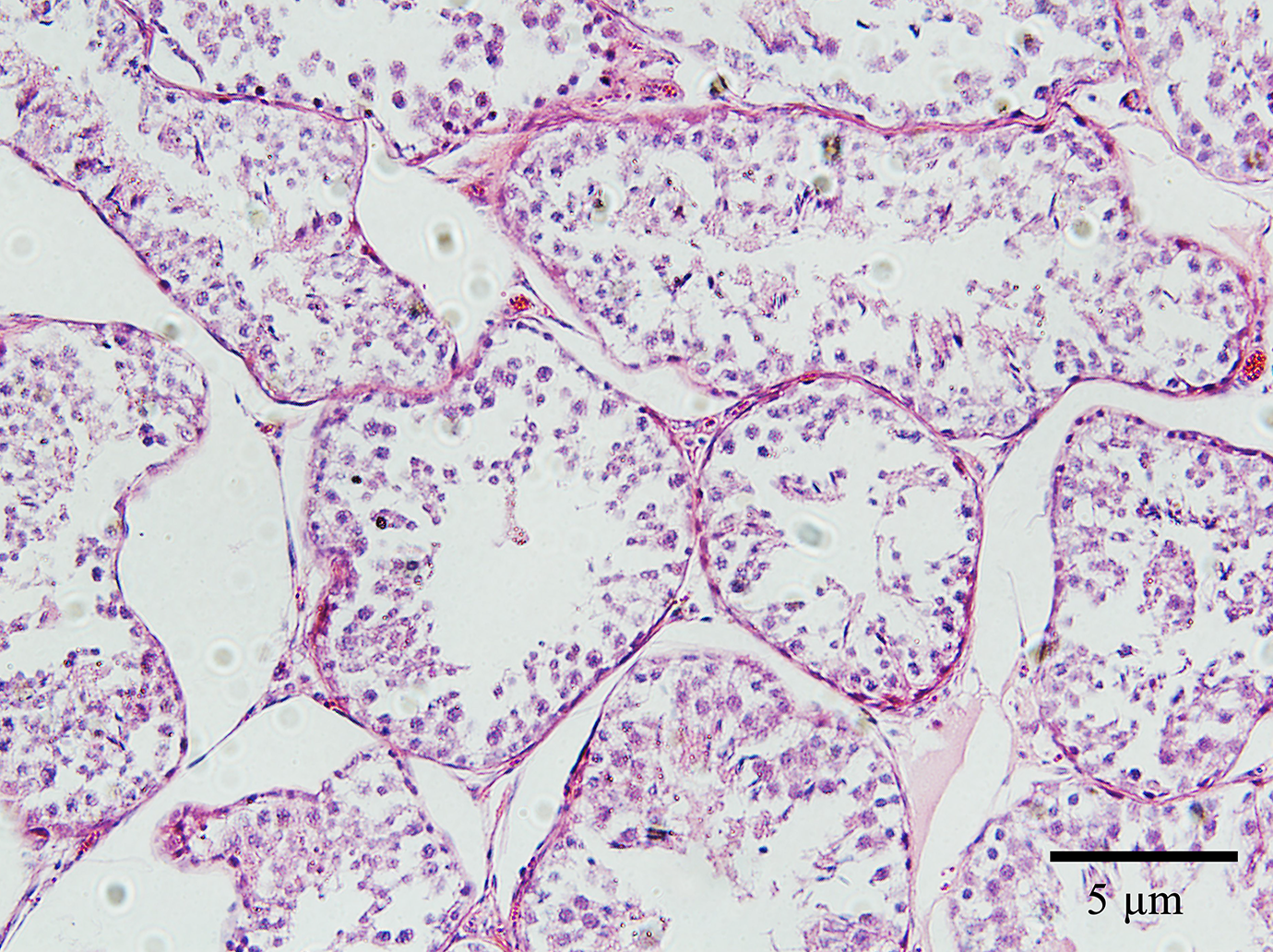

Supplement: Supplementary Data Sheet 2 — Microscopy images. [file Data_Sheet_2.zip › Microscopy images/HE staining/8 months VA.tif]

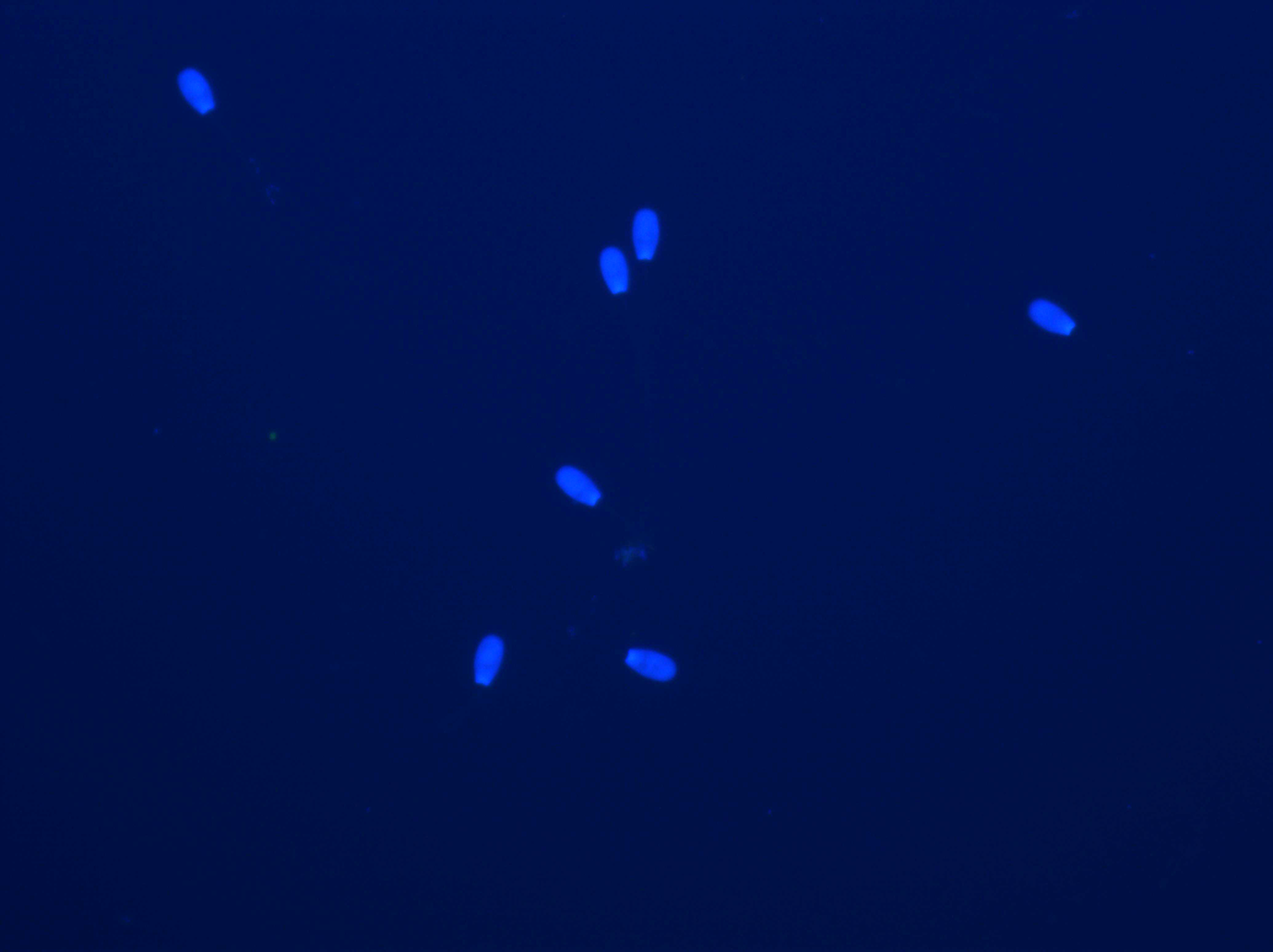

Supplement: Supplementary Data Sheet 2 — Microscopy images. [file Data_Sheet_2.zip › Microscopy images/IF/Control.DAPI.tif]

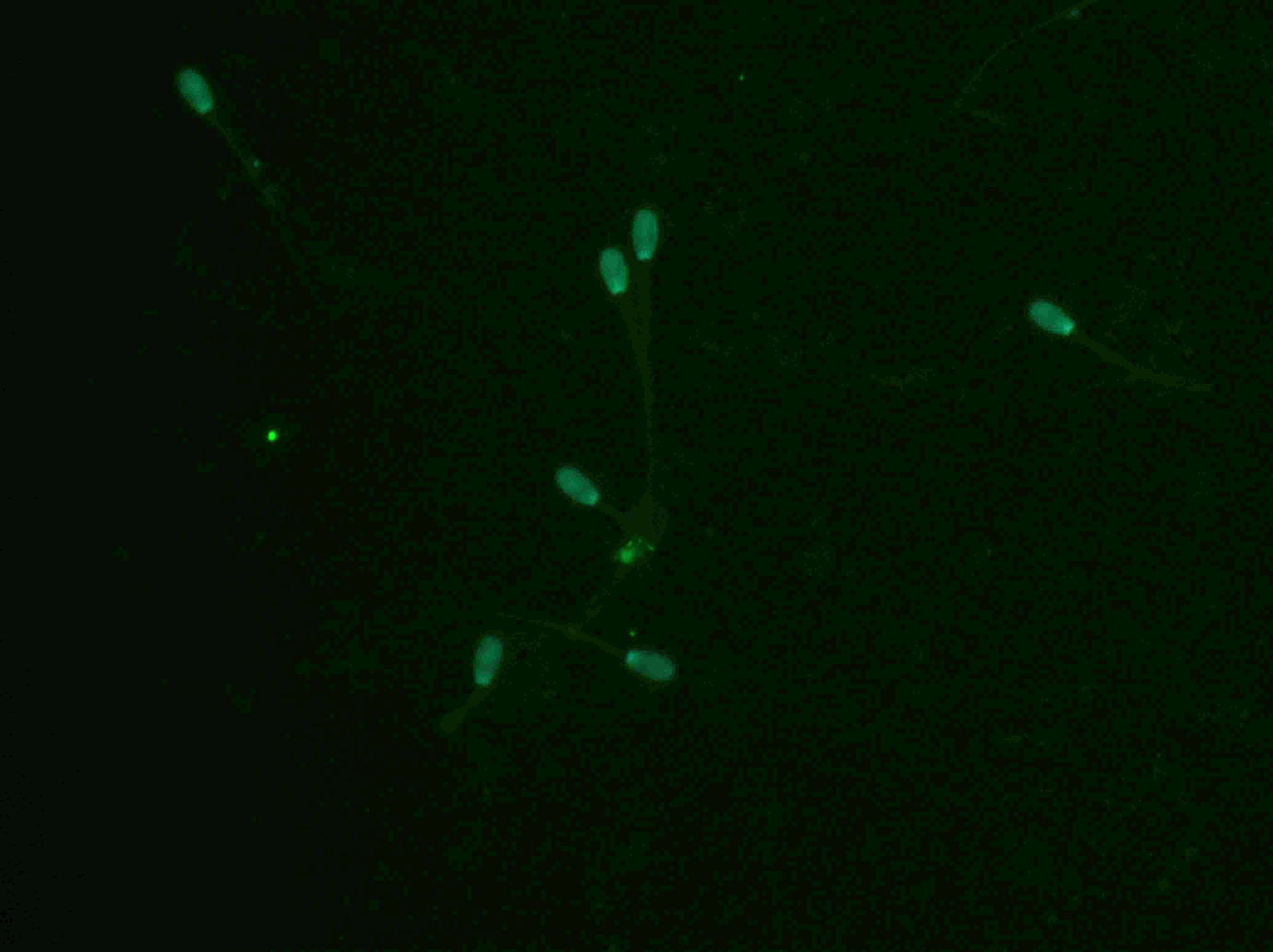

Supplement: Supplementary Data Sheet 2 — Microscopy images. [file Data_Sheet_2.zip › Microscopy images/IF/Control.GLUT3.tif]

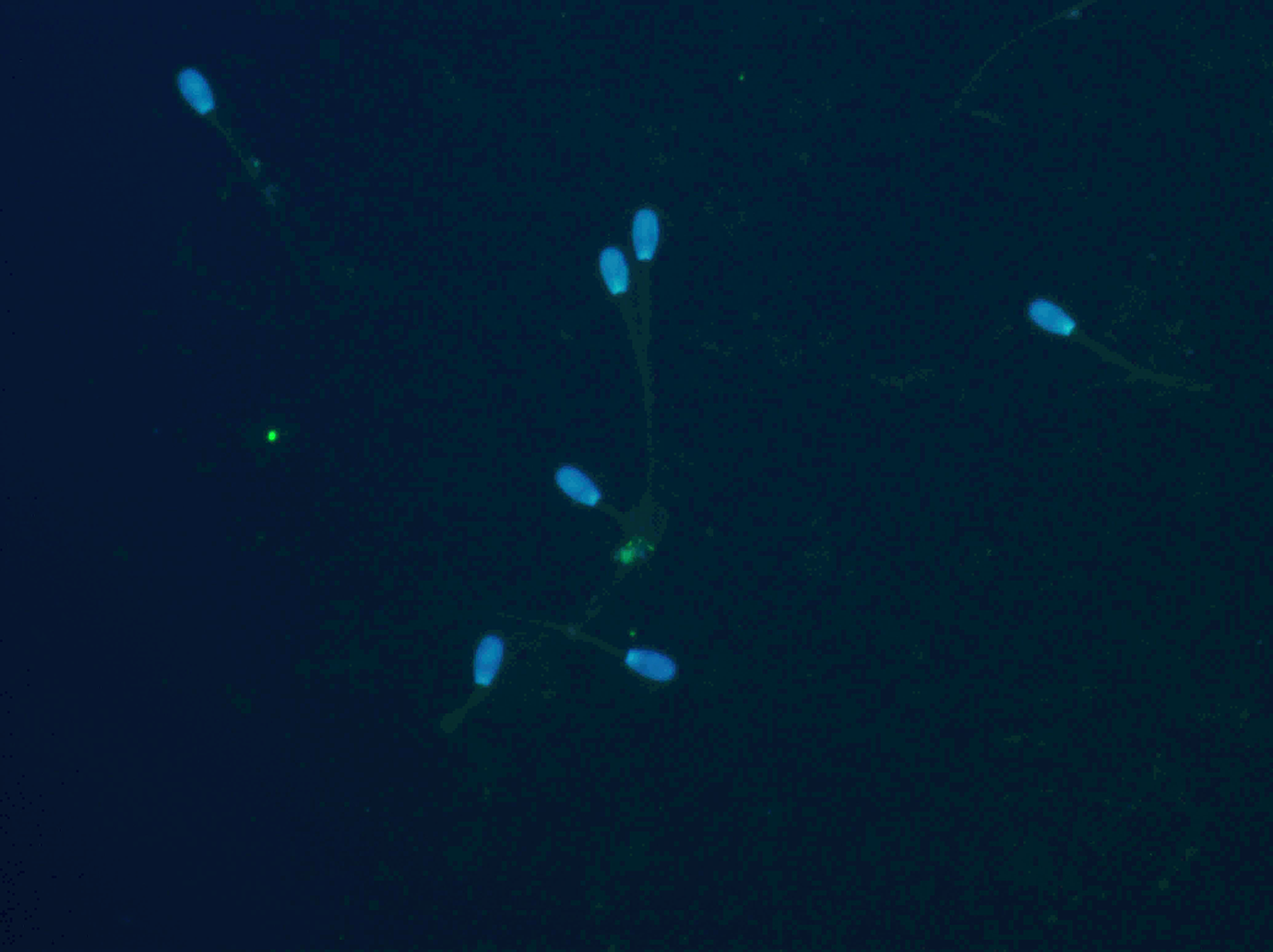

Supplement: Supplementary Data Sheet 2 — Microscopy images. [file Data_Sheet_2.zip › Microscopy images/IF/Control.Merge.tif]

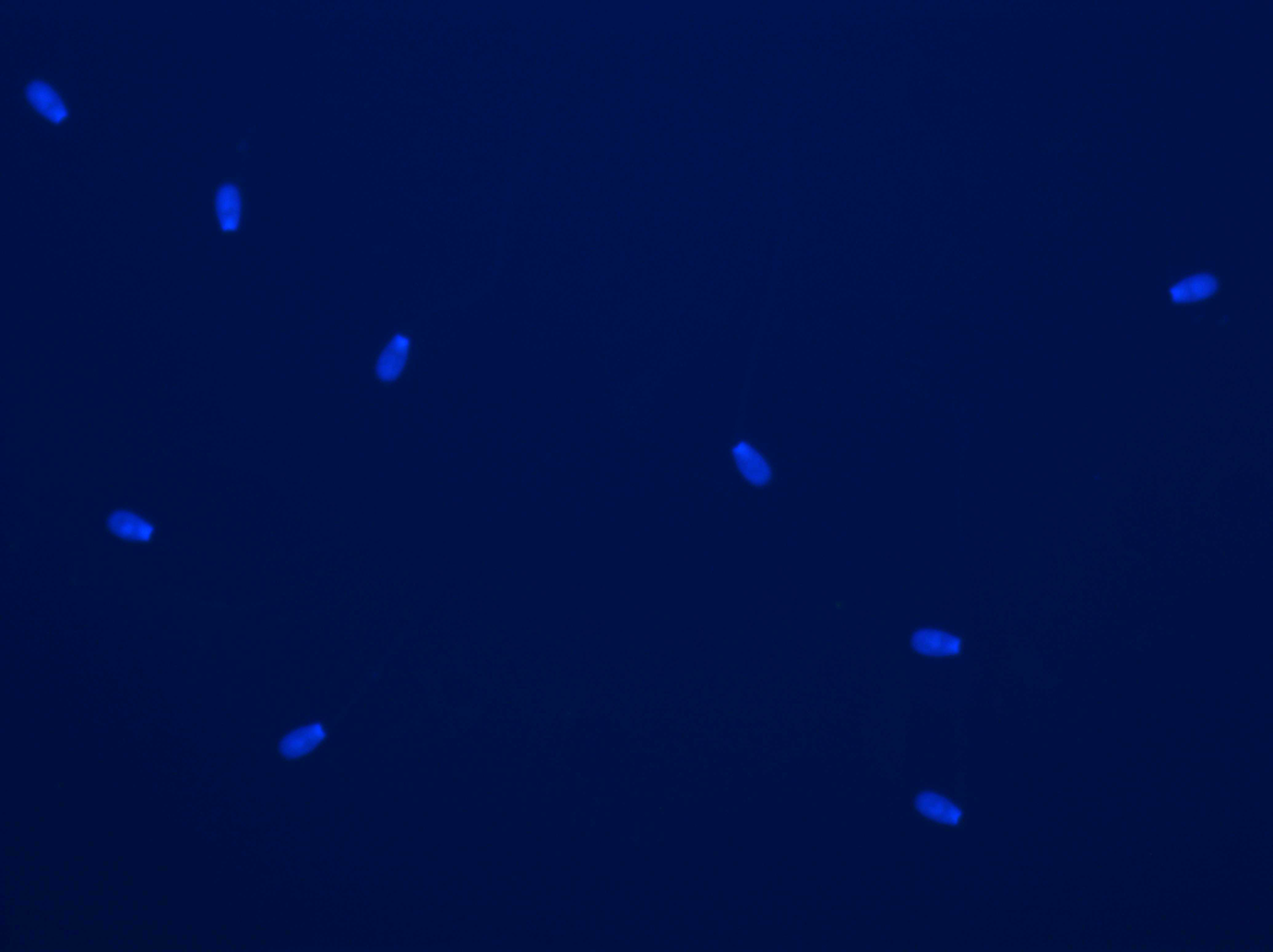

Supplement: Supplementary Data Sheet 2 — Microscopy images. [file Data_Sheet_2.zip › Microscopy images/IF/Negative control.DAPI.tif]

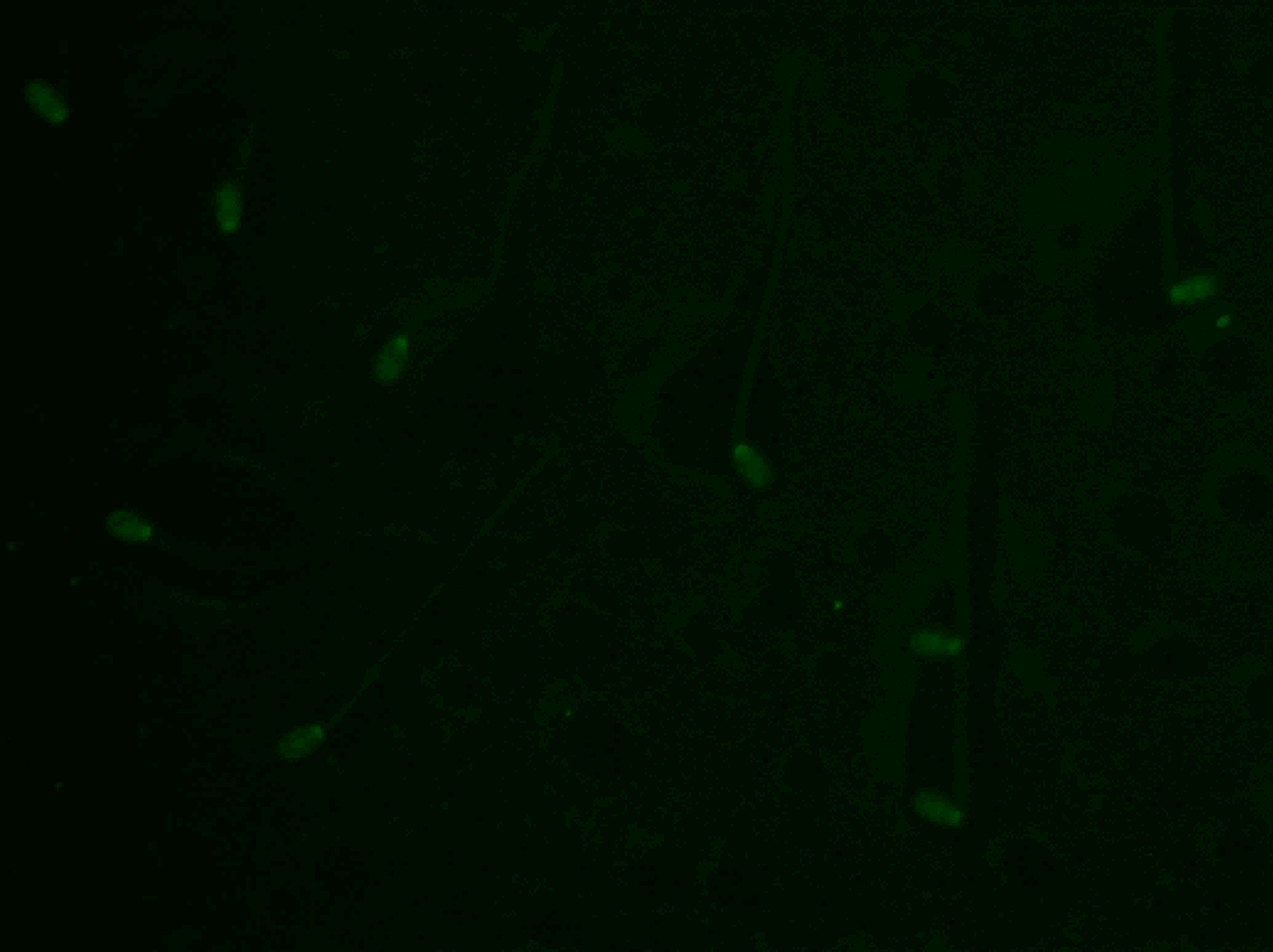

Supplement: Supplementary Data Sheet 2 — Microscopy images. [file Data_Sheet_2.zip › Microscopy images/IF/Negative control.GLUT3.tif]

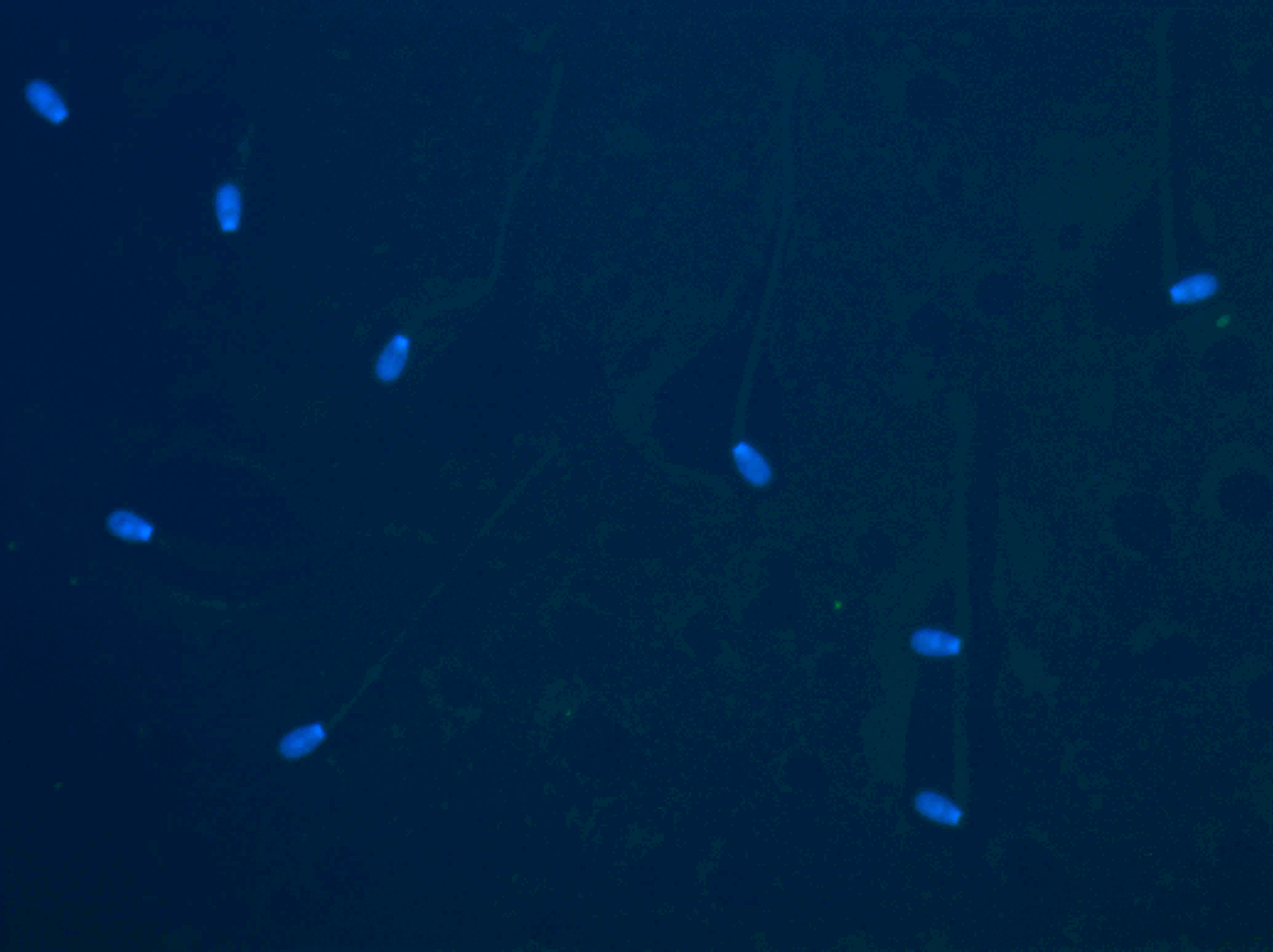

Supplement: Supplementary Data Sheet 2 — Microscopy images. [file Data_Sheet_2.zip › Microscopy images/IF/Negative control.Merge.tif]

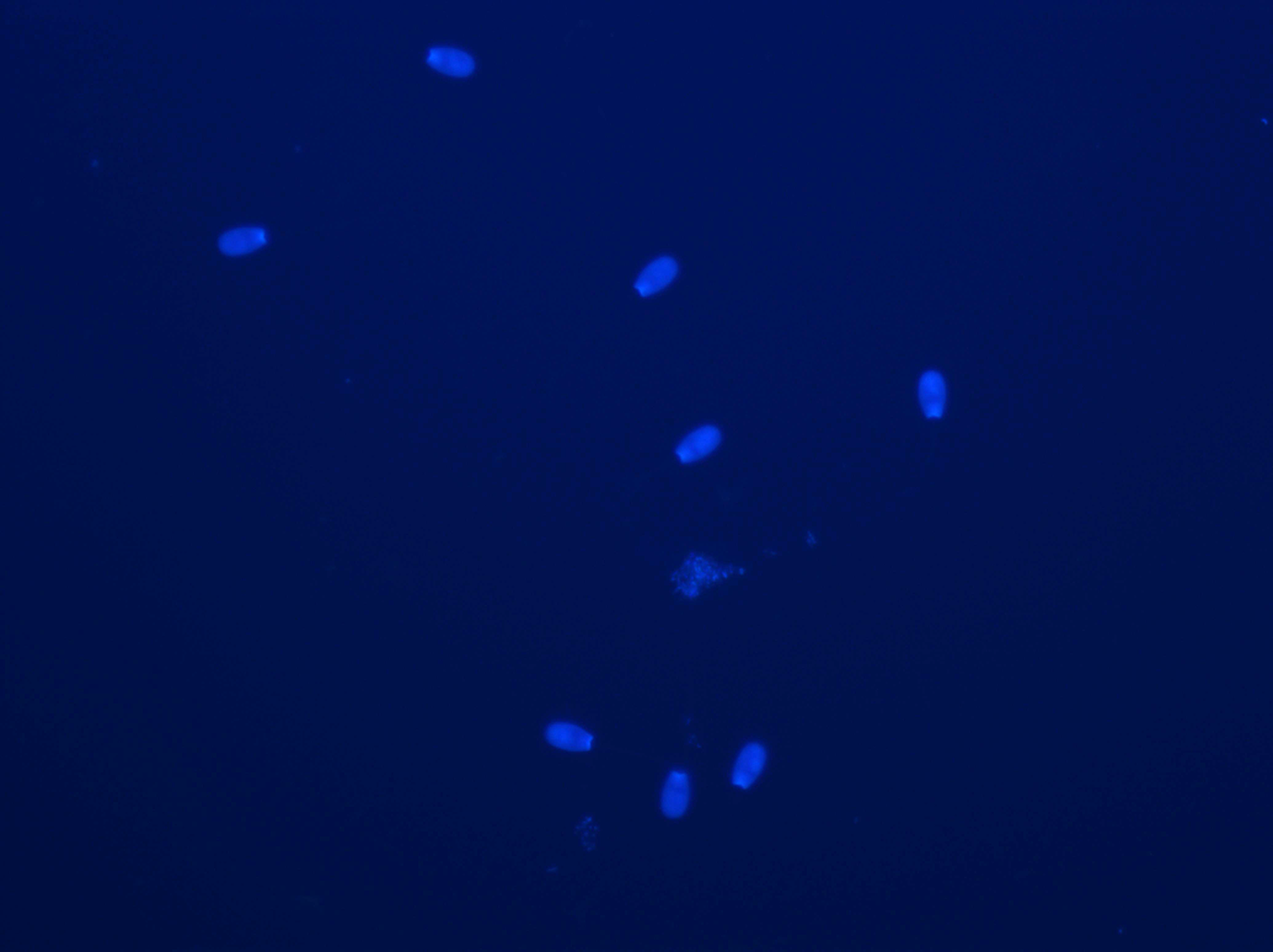

Supplement: Supplementary Data Sheet 2 — Microscopy images. [file Data_Sheet_2.zip › Microscopy images/IF/VA.DAPI.tif]

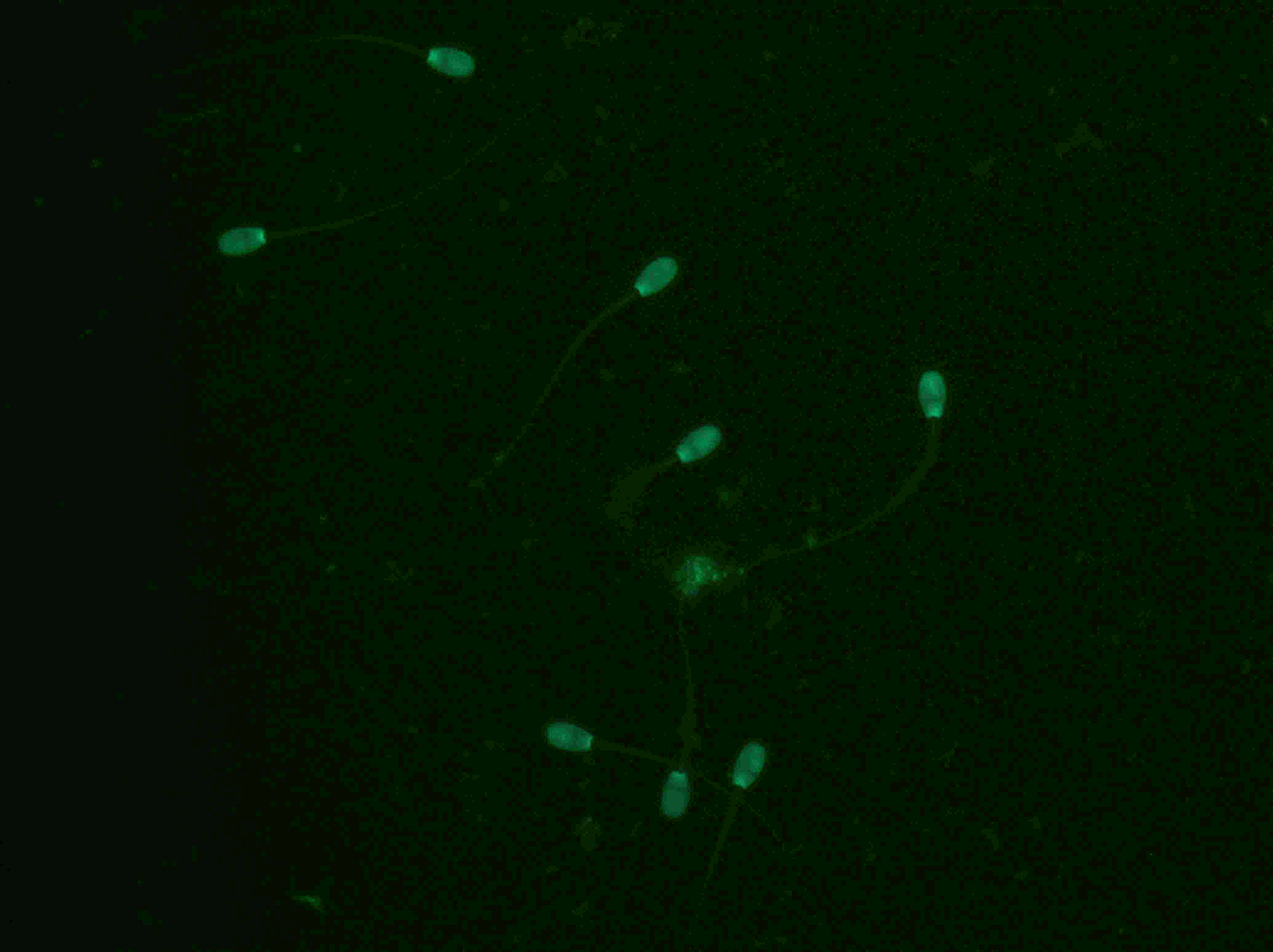

Supplement: Supplementary Data Sheet 2 — Microscopy images. [file Data_Sheet_2.zip › Microscopy images/IF/VA.GLUT3.tif]

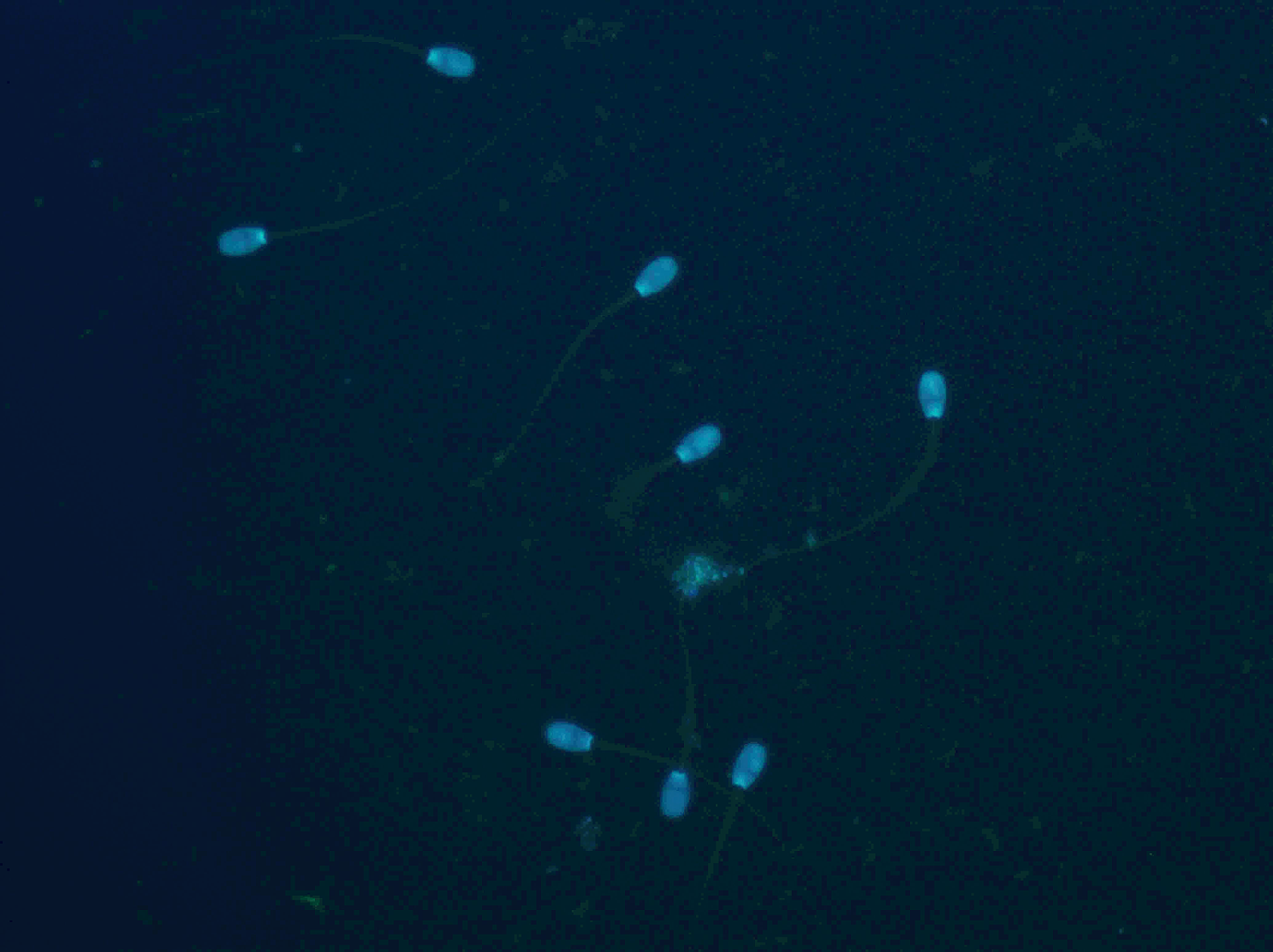

Supplement: Supplementary Data Sheet 2 — Microscopy images. [file Data_Sheet_2.zip › Microscopy images/IF/VA.Merge.tif]
